# Supplementary material for: Proteomic characterization of intrahepatic cholangiocarcinoma identifies risk-stratifying subgroups and EIF4A1 as a therapeutic target
Source: Nat Commun. 2026 Mar 23;17:2741. doi: 10.1038/s41467-026-70817-1 (PMC13013968; doi:10.1038/s41467-026-70817-1)
Supplement: Supplementary file 1 — Supplementary Information [file 41467_2026_70817_MOESM1_ESM.pdf]

Supplementary Figures

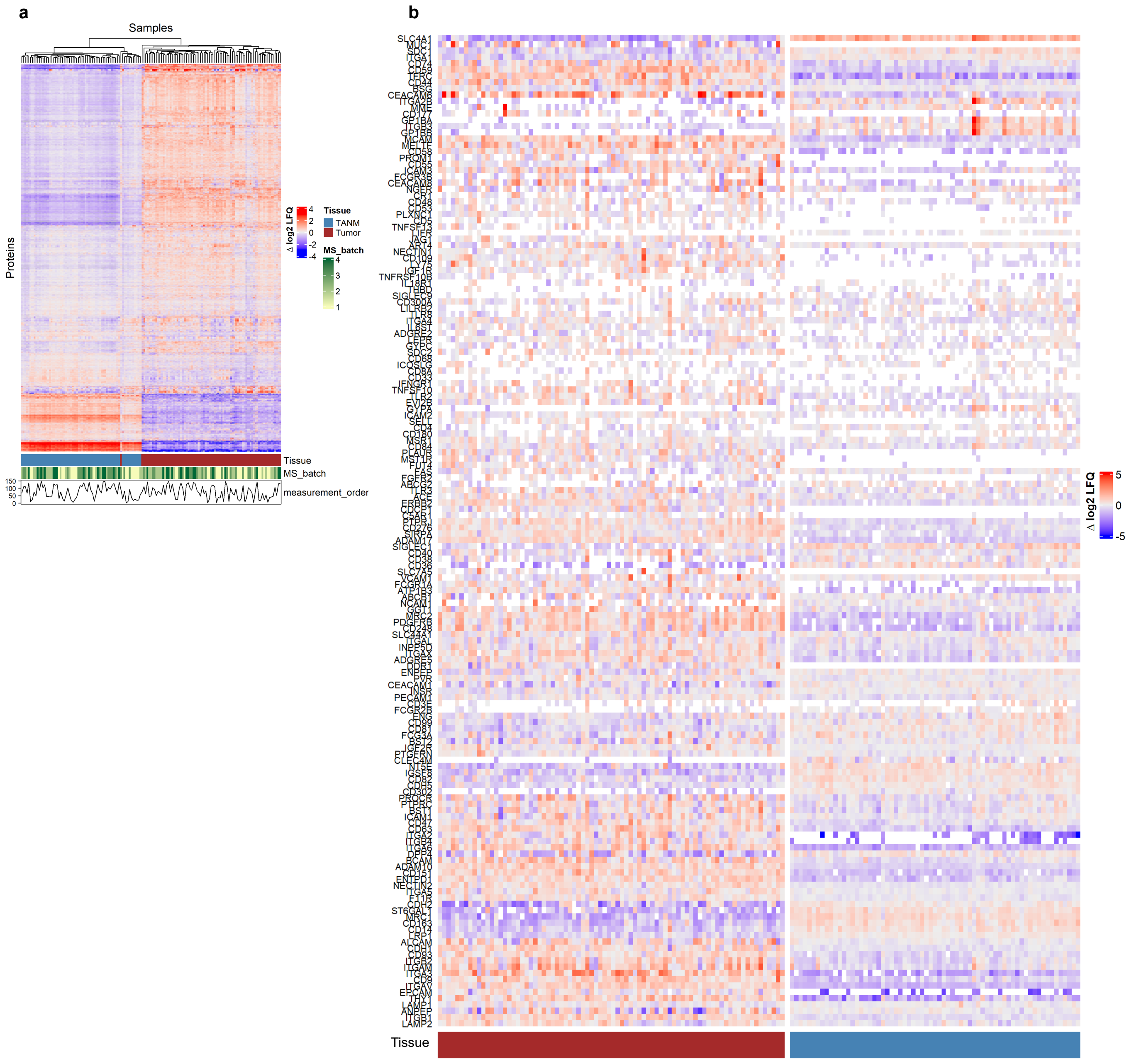

Supplementary Figure 1 – Heatmaps of protein abundance in tumor and TANM samples. The color scale represents row-wise median-normalized log2-transformed intensities. The red bar indicates tumor (n = 80), the blue bar indicates TANM tissue (n = 69). A) Heatmap comparing the hierarchically clustered cohort to tissue type, measurement batch and measurement order. B) Heatmap of immunity-related marker proteins. Source data are provided as a Source Data file.

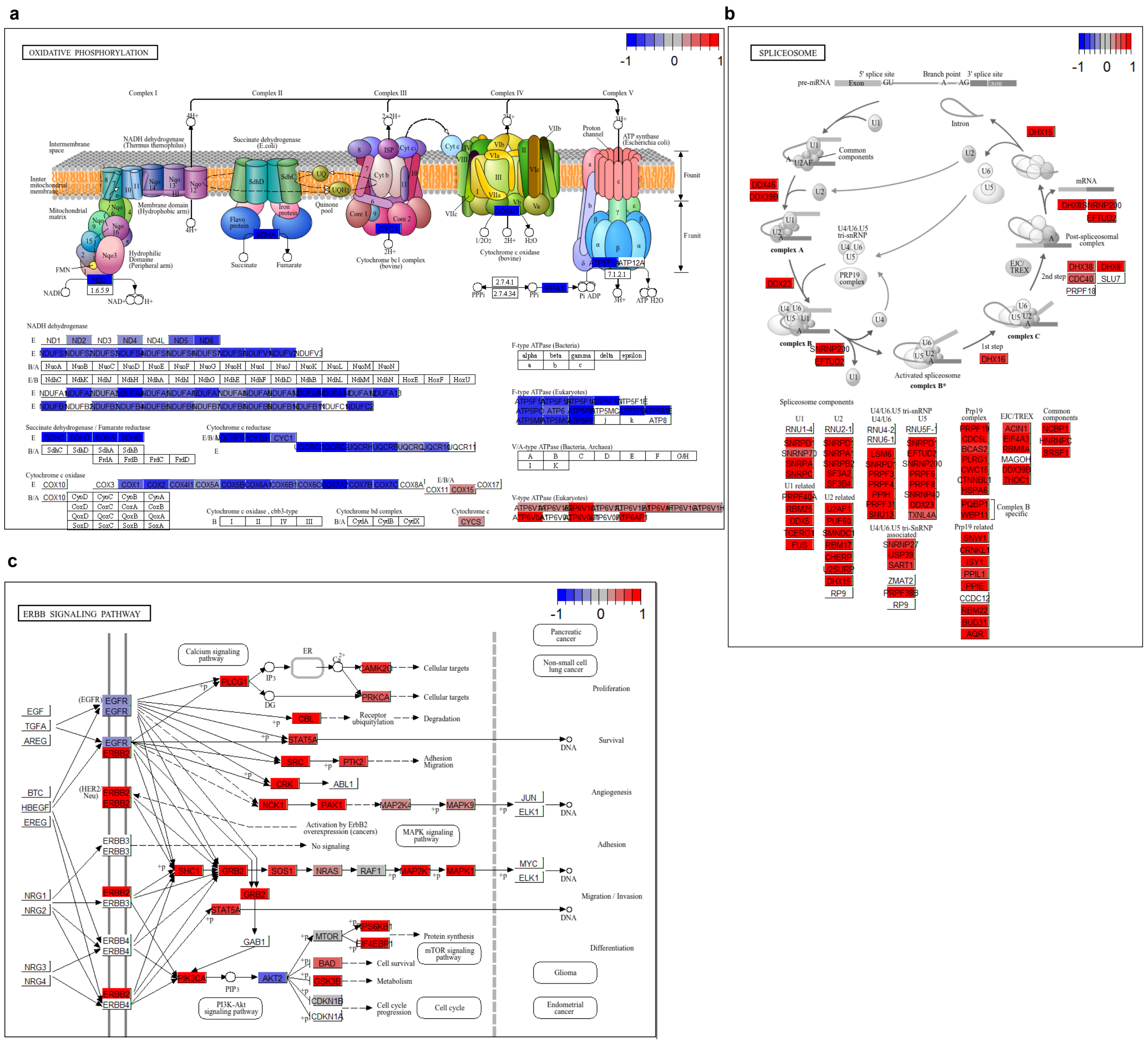

Supplementary Figure 2 - KEGG enrichment tumor vs. TANM.
Selected KEGG enrichment results of Limma tumor vs. TANM, generated with Pathview[1]. Terms enriched in tumor samples are shown in red (n = 80), and those enriched in TANM samples are shown in blue (n = 69). Color gradient indicates normalized protein abundance change. A) Oxidative Phosphorylation. B) Spliceosome. C) ERBB Signaling Pathway.

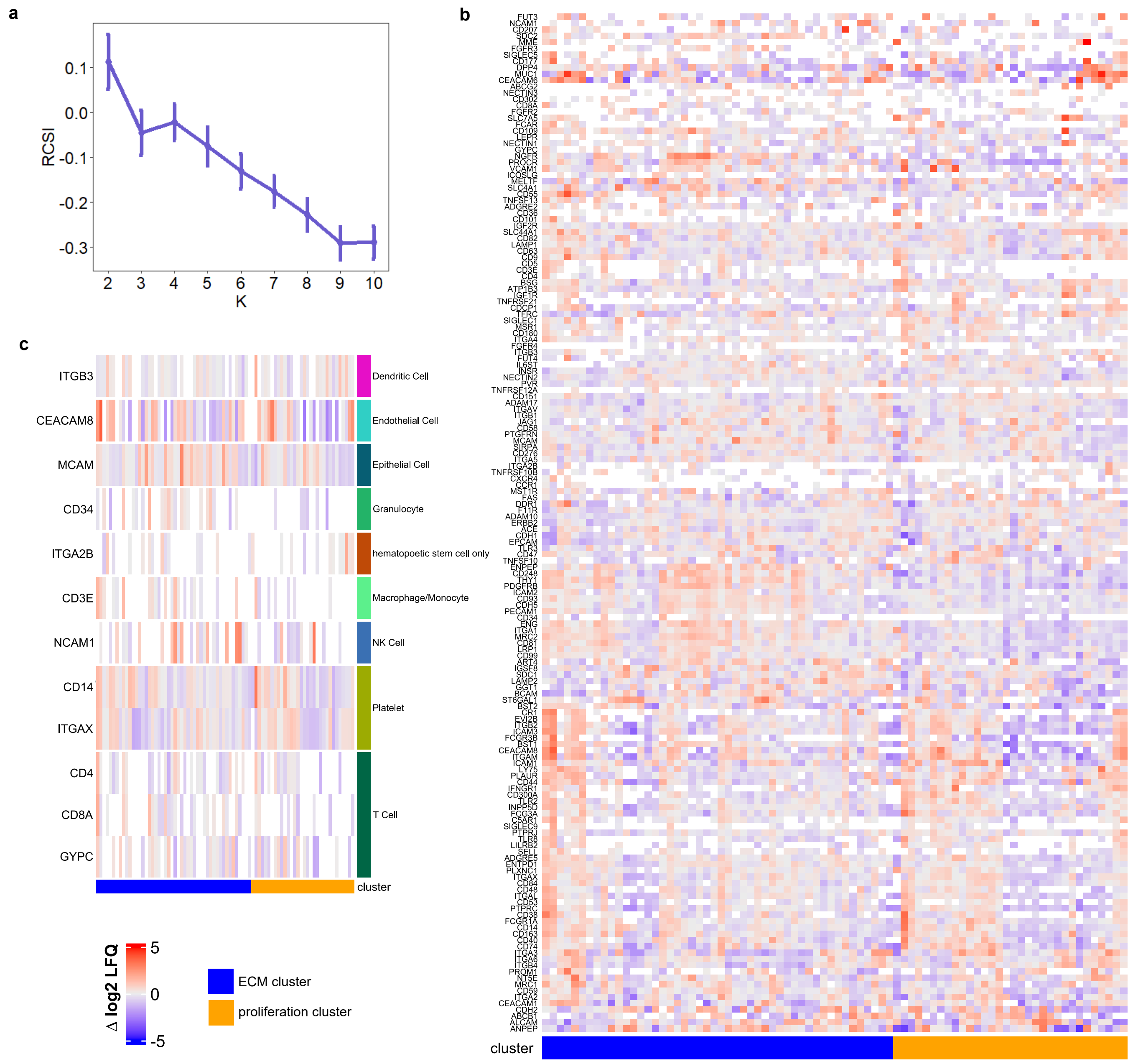

Supplementary Figure 3 – Clustering of tumor samples and heatmaps of immunity-related protein abundance.

A) Monte-Carlo simulation result of clustered tumor samples (n = 80). Potential numbers of subclusters k can be selected through the Relative Cluster Stability Index (RCSI). B & C) Heatmaps of immune cell (B) and immunity-related (C) marker proteins. Blue bar indicates ECM cluster (n = 48), orange bar indicates proliferation cluster (n = 32). The color scale represents row-wise median-normalized log2-transformed intensities.

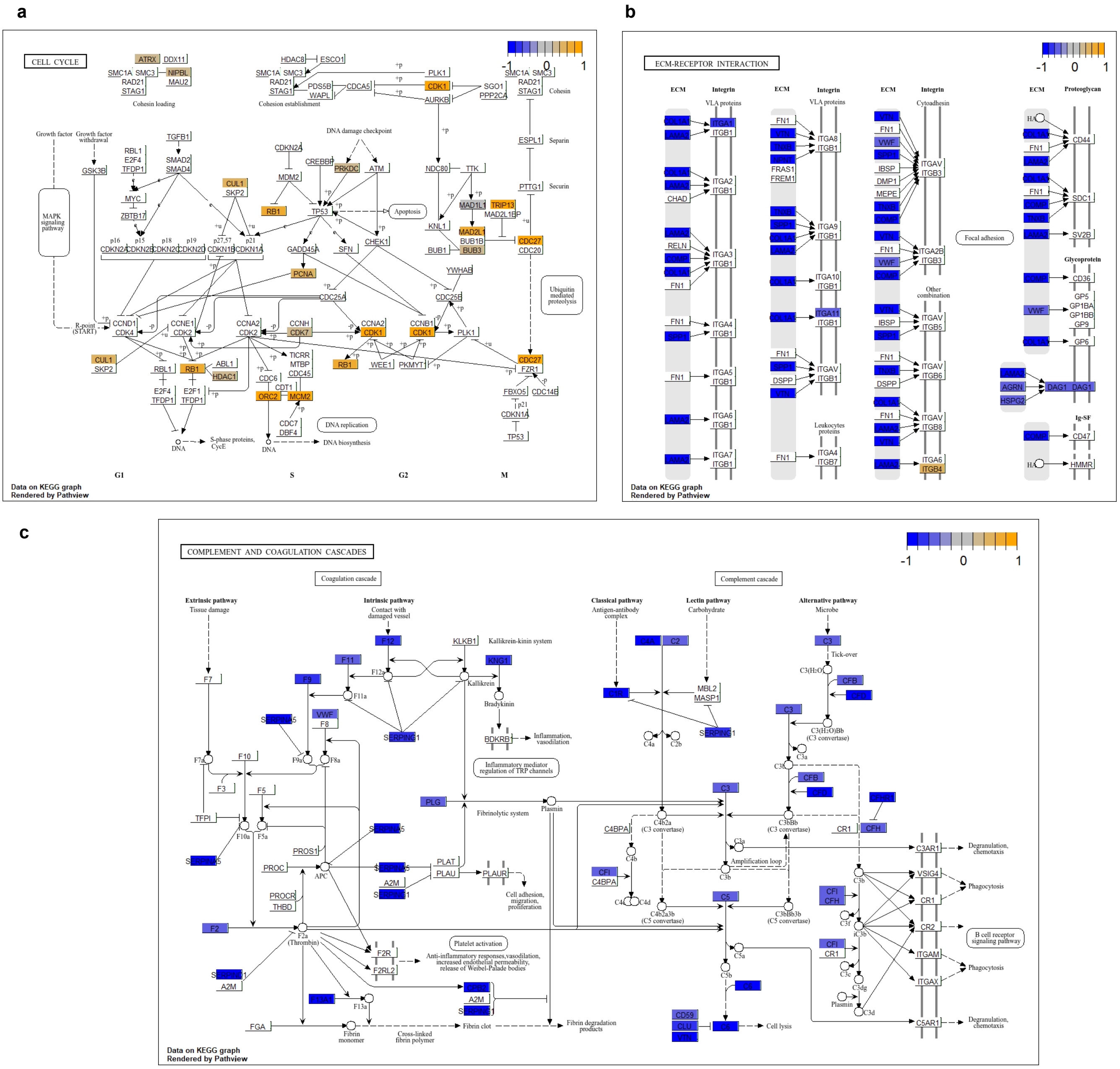

Supplementary Figure 4 - KEGG enrichment ECM vs. proliferation cluster.
Selected KEGG enrichment results of Limma ECM cluster vs. proliferation cluster, generated with Pathview[1]. Terms enriched in the ECM cluster (n = 48) are shown in blue, and those enriched in the proliferation cluster (n = 32) are shown in blue. Color gradient indicates normalized protein abundance change. A) Cell Cycle. B) ECM-Receptor Interaction. C) Complement and Coagulation Cascades.

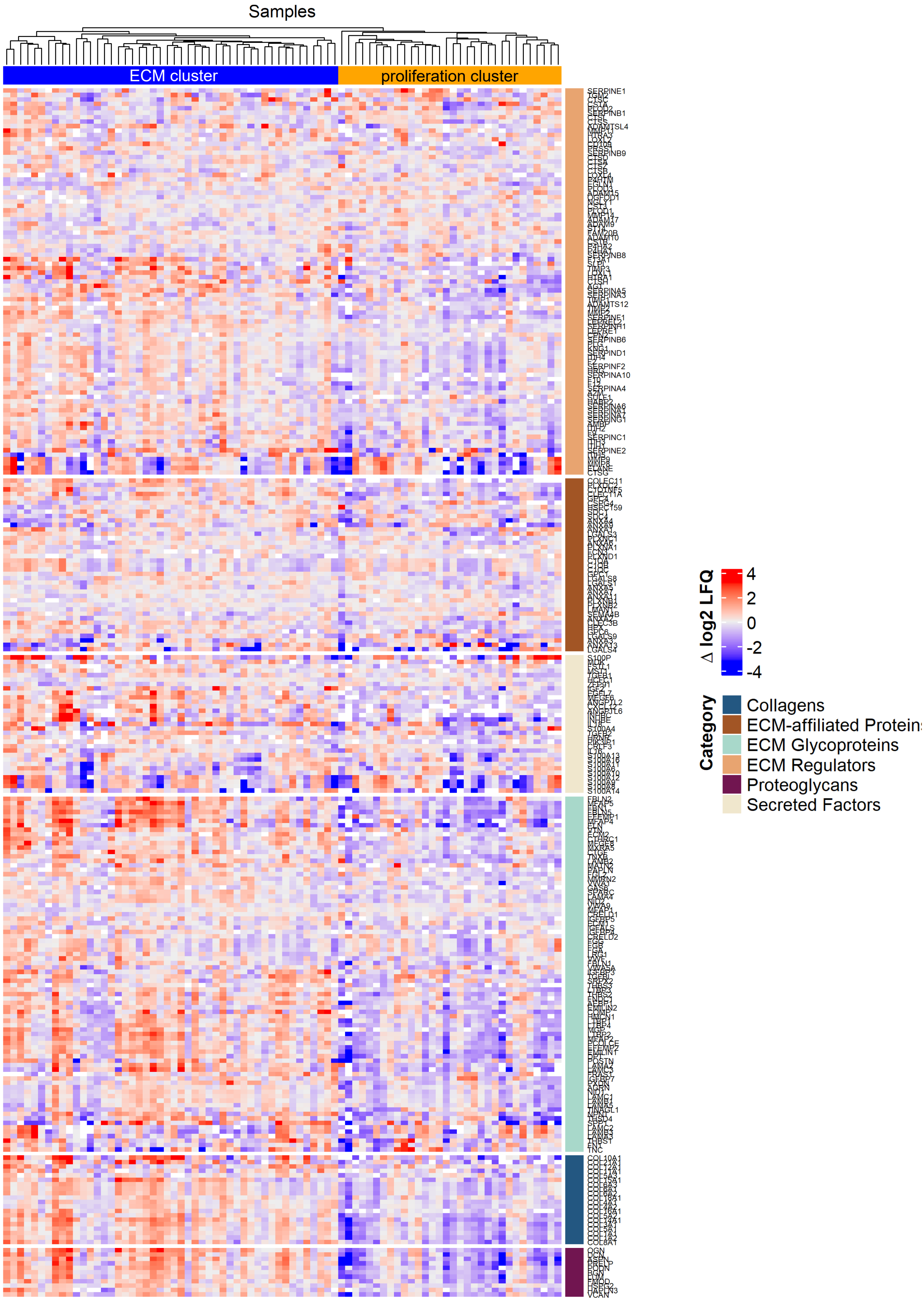

Supplementary Figure 5 – ECM protein expression: Heatmap of ECM protein expression in hierarchically clustered tumor samples.

Blue bar indicates ECM cluster (n = 48), orange bar indicates proliferation cluster (n = 32). Vertical bars indicate ECM category. The color scale represents row-wise median-normalized log2-transformed intensities. Source data are provided as a Source Data file.

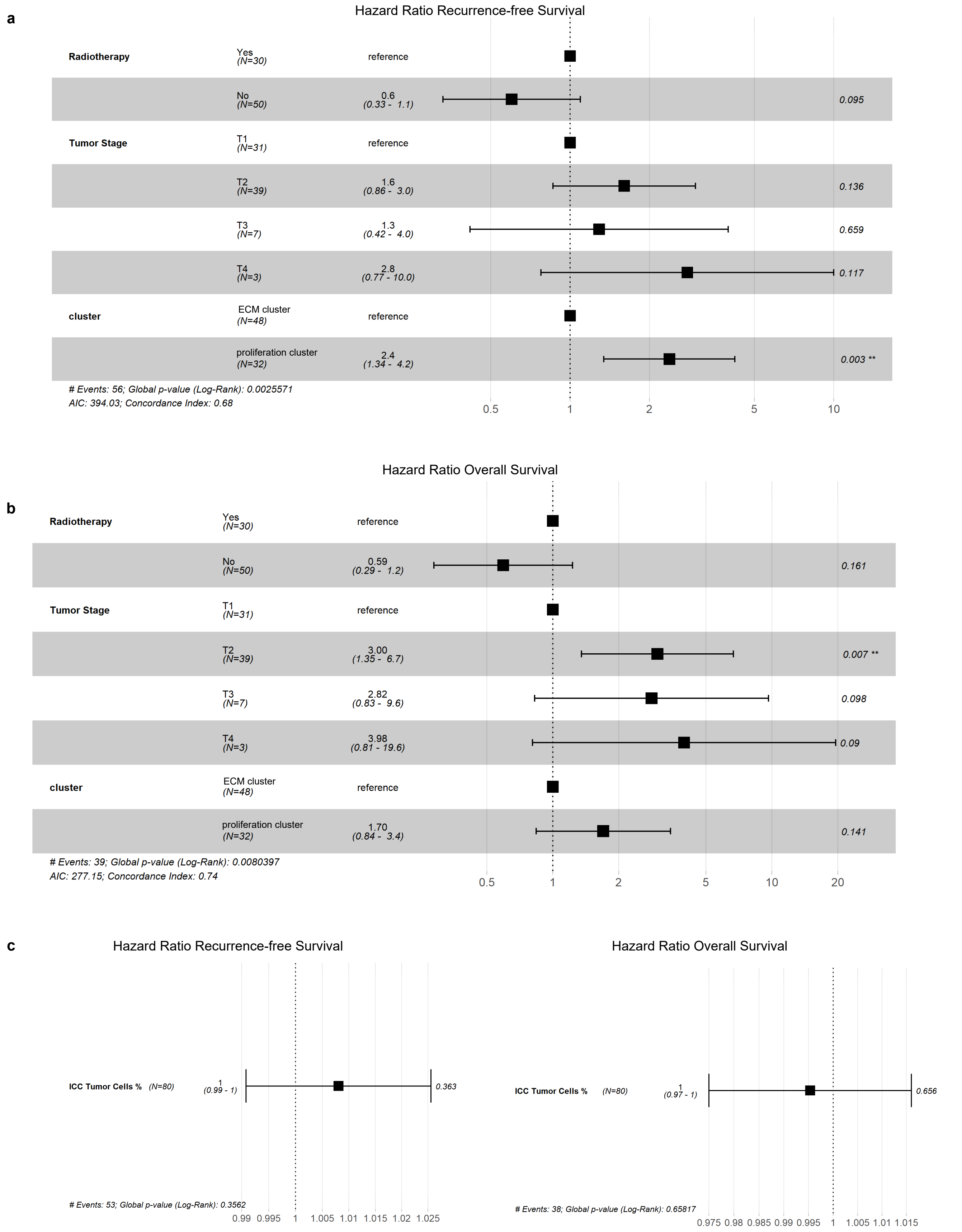

Supplementary Figure 6 – Multivariable Cox Proportional Hazards Model (CPHM) - Hazard Ratios (incl. 95% confidence intervals) for selected parameters.

A) CPHM for recurrence-free survival including radiotherapy, tumor stage, and cluster assignment as covariates, p-values are two-sided. B) CPHM for overall survival including radiotherapy, tumor stage, and cluster assignment as covariates, p-values are two-sided. C) Impact of tumor cell content [%] on left) recurrence-free survival and right) overall survival. Source data are provided as a Source Data file.

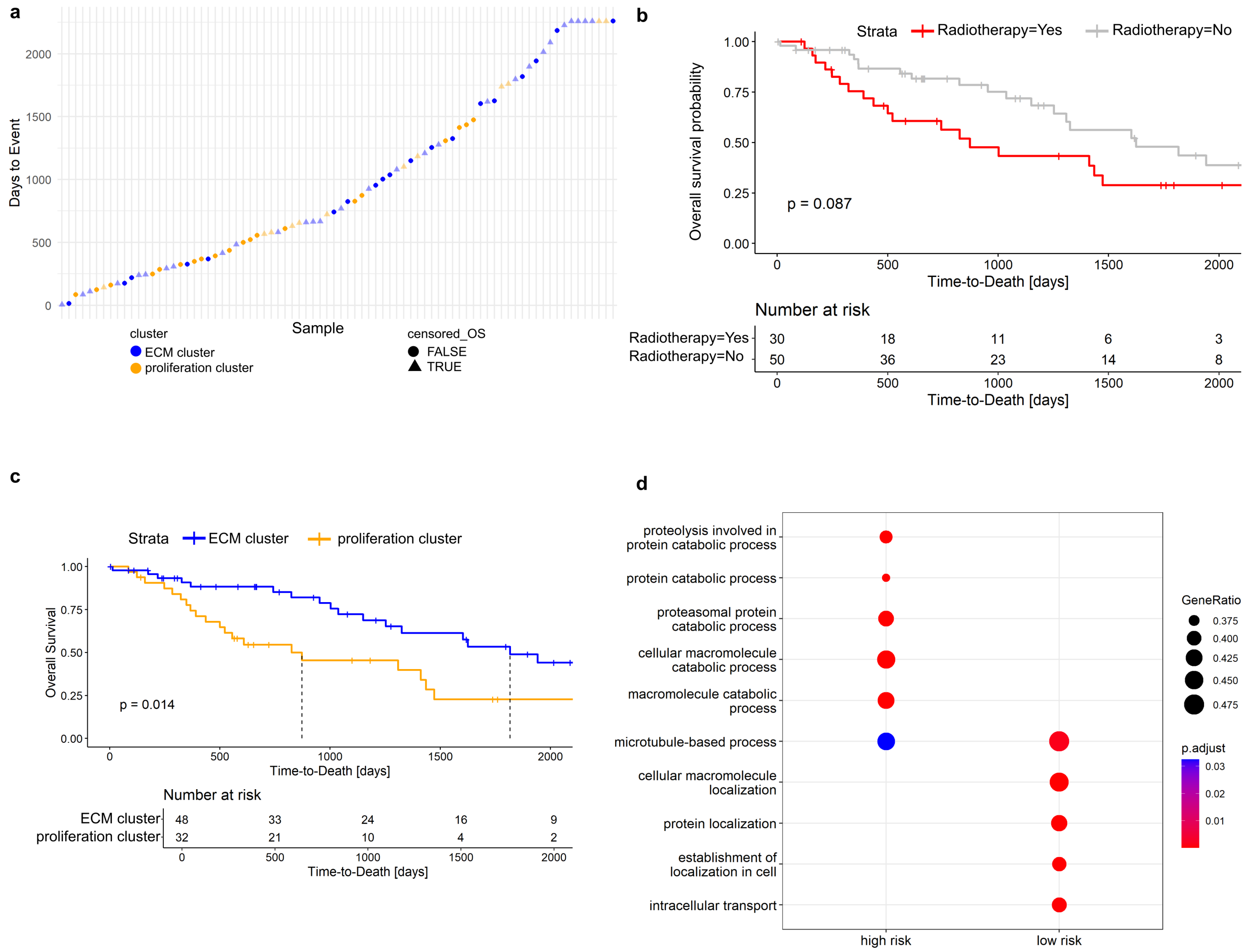

Supplementary Figure 7 - Statistical analysis of overall survival data.
A) Overall survival distribution across the cohort (n = 80). B) Kaplan-Meier plot incl. log-rank test comparing overall survival probability between patients treated with or without radiotherapy. C) Kaplan-Meier plot incl. log-rank test comparing overall survival between clusters. D) Gene set enrichment analysis (GSEA) of Cox Proportional Hazards Model results. Source data are provided as a Source Data file.

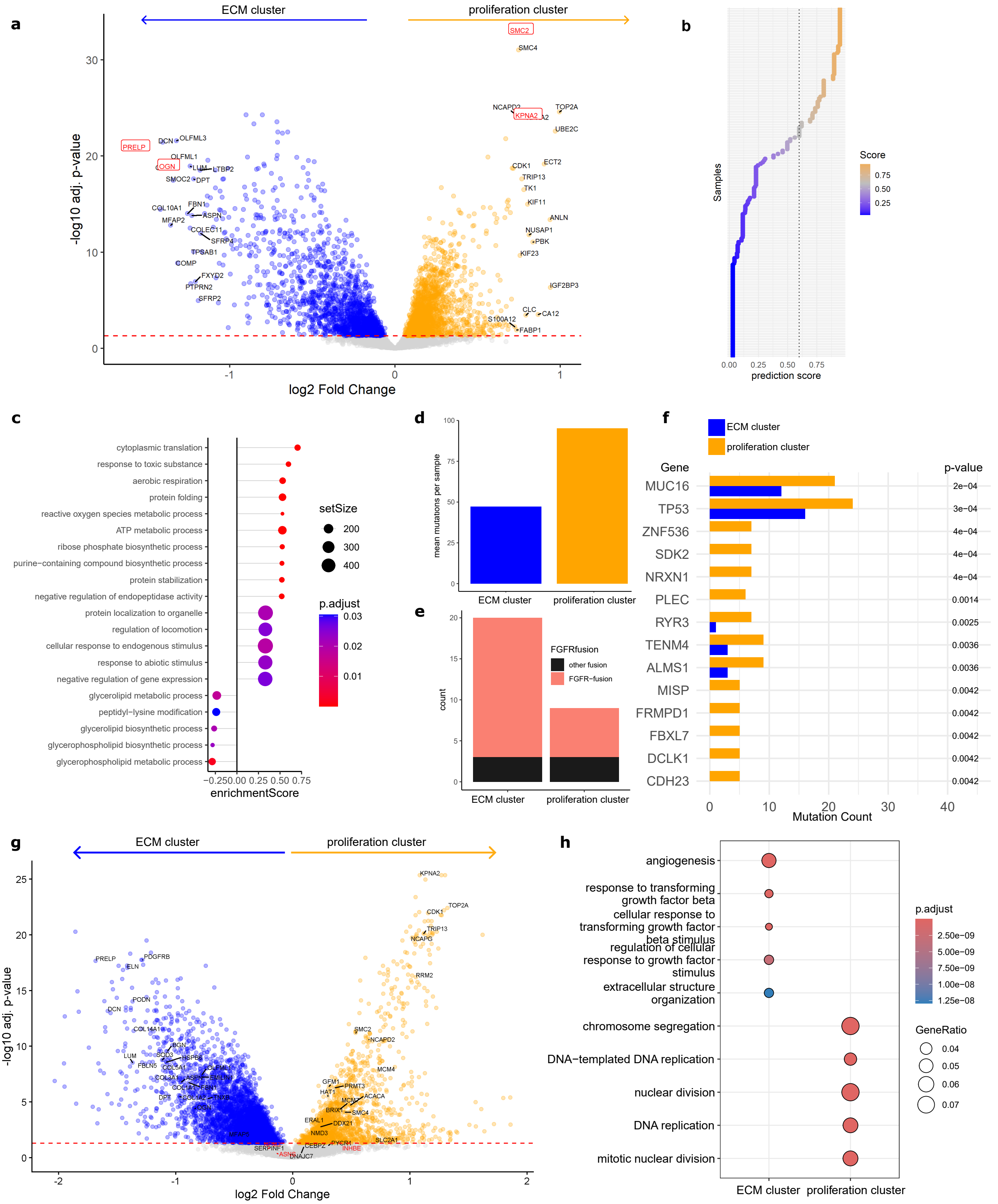

Supplementary Figure 8 – Tumor clusters in the FU-ICC cohort.
A) Volcano plot of differentially expressed proteins between ECM (n = 141) and proliferation cluster (n = 73) (log2-fold changes from limma; Benjamini-Hochberg (BH)-adjusted significant proteins in colors, red labels = classifier selection). B) Prediction score and sample classification. C) GO Gene Set Enrichment Analysis for ECM vs. proliferation cluster. D) Mean mutations per sample. E) Fusion counts. F) Genes with significantly different mutation counts between clusters (two-sided Fisher's exact tests, p-value adjustment via Benjamini-Hochberg). G) Volcano plot of differentially expressed genes, with top proteomic hits labeled and discordant directions marked in red (log2-fold changes from limma; BH-adjusted significant proteins in colors). H) Over representation analysis of differentially expressed genes (FDR-corrected permutation test).

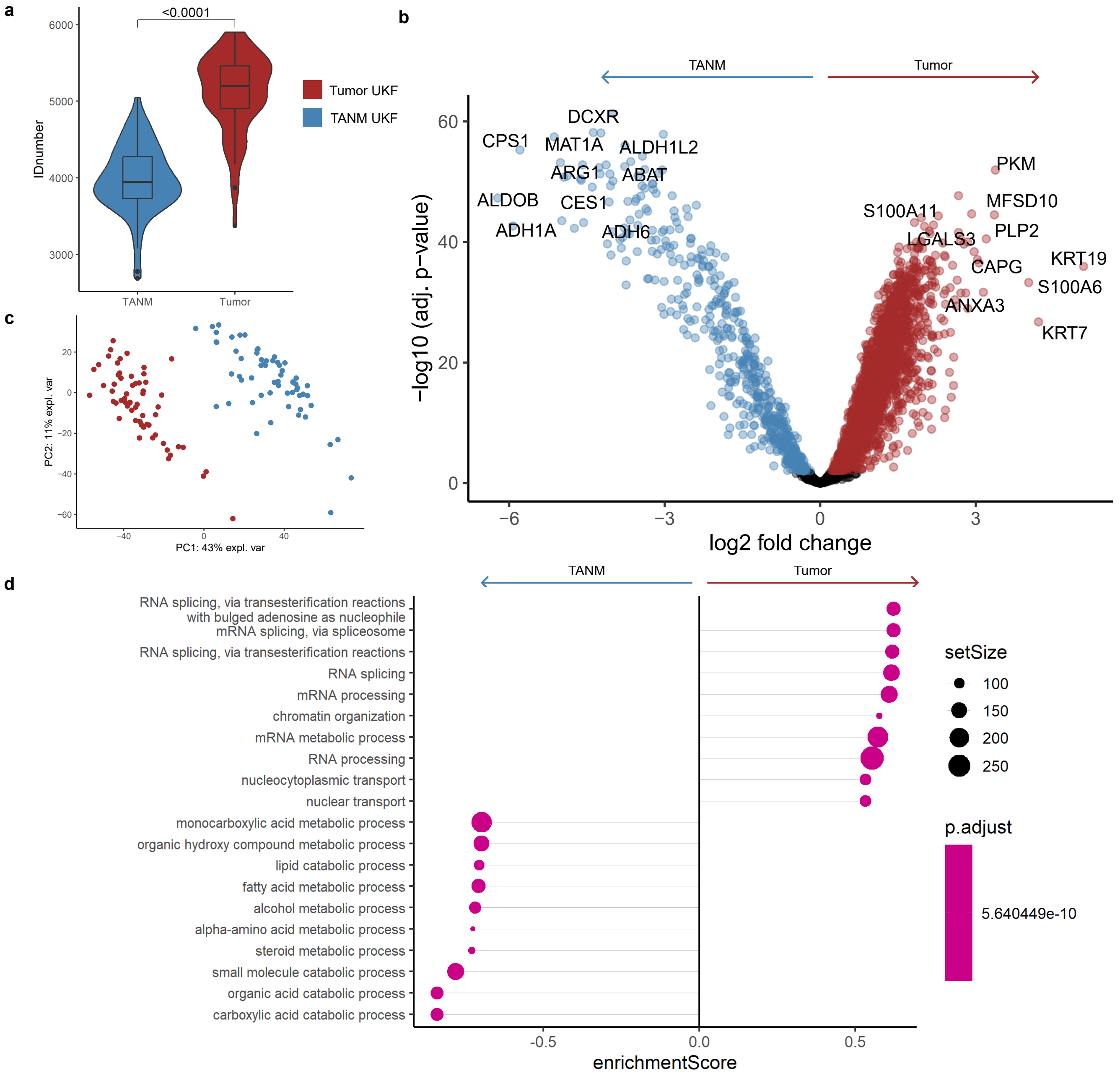

Supplementary Figure 9 - Tumor vs. TANM tissue in the UKF-ICC cohort.
Blue color indicates TANM tissue (n = 61), red color indicates tumor tissue (n = 62). A) Protein ID numbers across all samples (n = 123) of the cohort compared between TANM and tumor tissue (unpaired t-test). B) Volcano plot of differentially expressed proteins between tumor and TANM tissues. Log2-fold changes are derived from an univariable linear regression model. Dots colored in red (tumor) or blue (TANM) lie above the Benjamini-Hochberg-adjusted significance threshold. P-values are two-sided. C) Principal Component Analysis (PCA) of the entire cohort. D) Gene Ontology gene set enrichment analysis (GSEA) of differentially regulated biological processes between tumor and TANM. Top 20 terms are shown. Significance of GSEA items was determined by a permutation test with false-discovery rate (FDR)-based multiple testing correction. Source data are provided as a Source Data file.

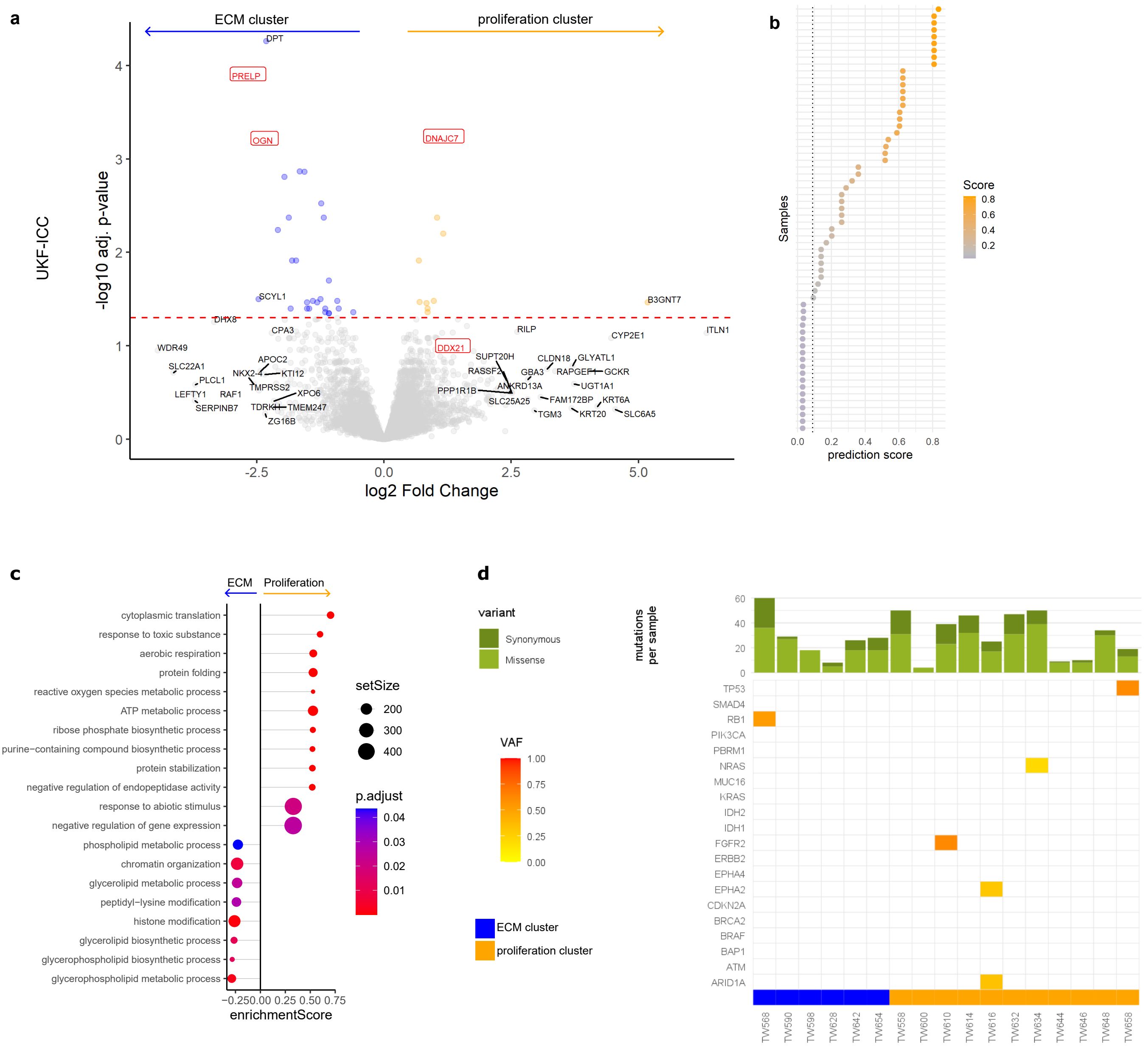

Supplementary Figure 10 – Tumor clusters in the UKF-ICC cohort.
A) Volcano plot of differentially expressed proteins between ECM (n = 18) and proliferation cluster (n = 42). Log2-fold changes are derived from an univariable linear regression model. Dots colored in blue (ECM cluster) or orange (proliferation cluster) lie above the Benjamini-Hochberg-adjusted significance threshold. P-values are two-sided. B) Prediction score and classification of tumor samples. C) Gene Ontology Gene Set Enrichment Analysis (GSEA) of Limma results ECM vs. proliferation cluster. D) Mean mutations per sample and mutations in selected genes. Source data are provided as a Source Data file.

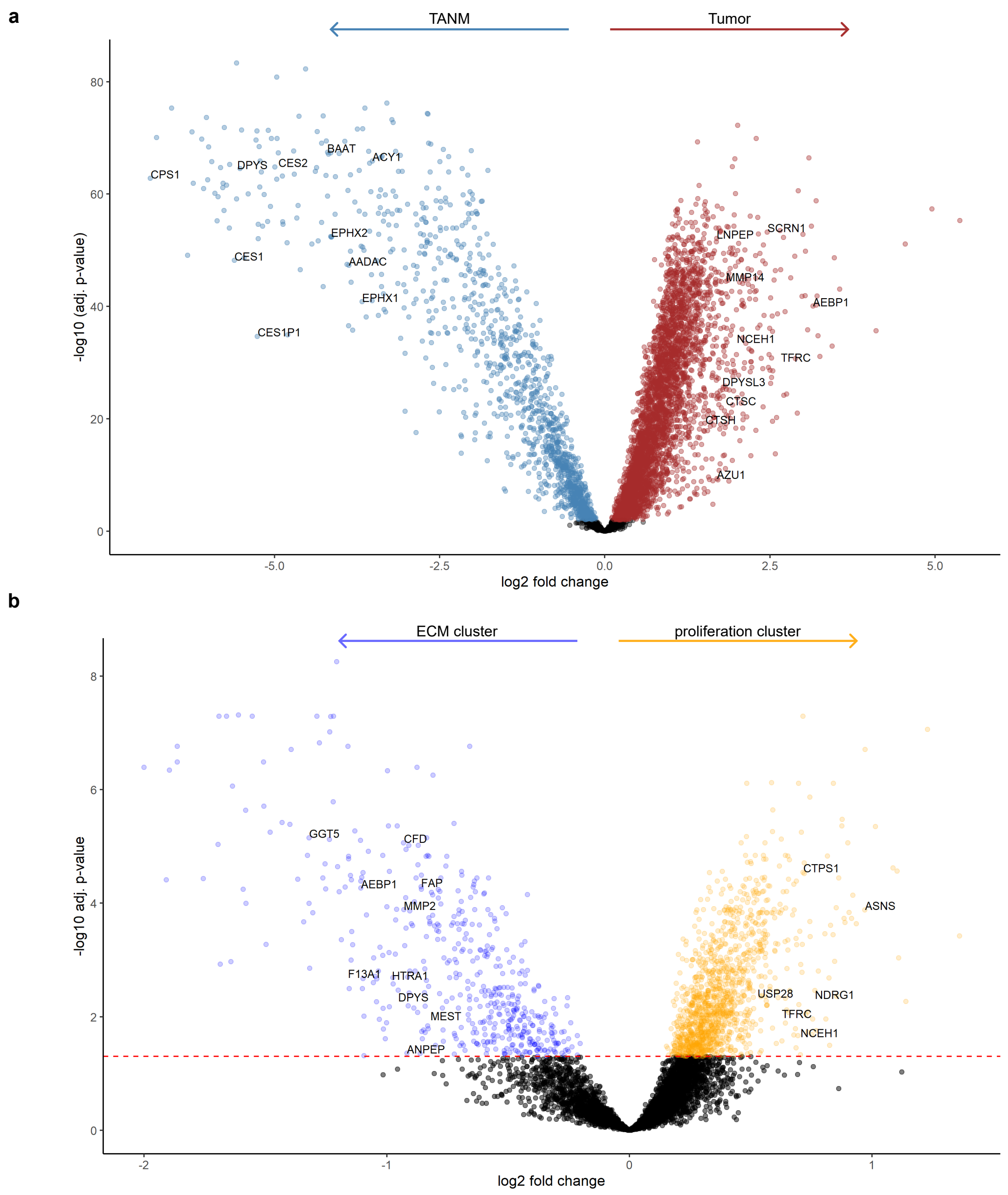

Supplementary Figure 11 - Volcano plots highlighting differentially abundant proteases in the MSKCC cohort.

Limma-based differential expression analysis, multiple testing correction via Benjamini-Hochberg. The top 10 proteases (identified via the MEROPS protease database) with the highest, significant log2 fold changes above 0.5 are named. A) Tumor (in red, n = 80) vs. TANM (in blue, n = 69). B) ECM cluster (in blue, n = 48) vs. proliferation cluster (in orange, n = 32). Source data are provided as a Source Data file.

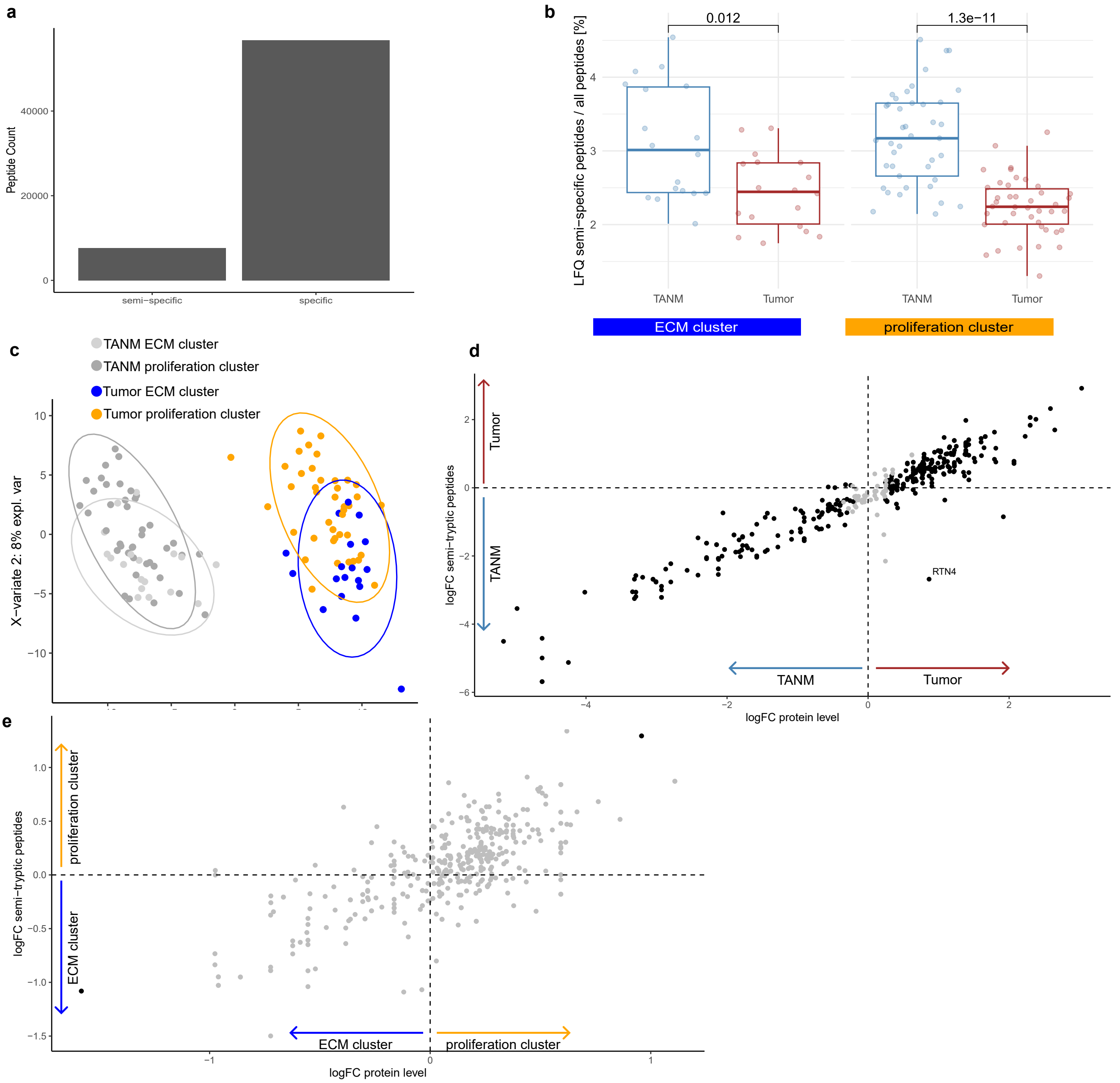

Supplementary Figure 12 - Semi-tryptic analysis of the UKF-ICC cohort.
A) Count of fully tryptic (specific) and semi-specific detected peptides across the cohort (n = 123). B) Boxplot indicating the ratio of semi-specific peptides among all detected peptides for tumor and TANM samples in the ECM and proliferation cluster. Unpaired, two-sided t-test. Boxplots show median (center line), interquartile range (IQR, extending from the 1st to the 3rd quartile, box), and 1.5 IQR (whiskers). C) Partial Least Squares-Discriminant Analysis (PLS-DA) of semi-specific peptide expression in both clusters and matching TANM tissues. Ovals indicate 95% confidence intervals. D) Correlation of logFC values between protein abundance and semi-tryptic peptide abundance in tumor vs. TANM tissue. E) Correlation of logFC values between protein abundance and semi-tryptic peptide abundance across ECM vs. proliferation clusters. Source data are provided as a Source Data file.

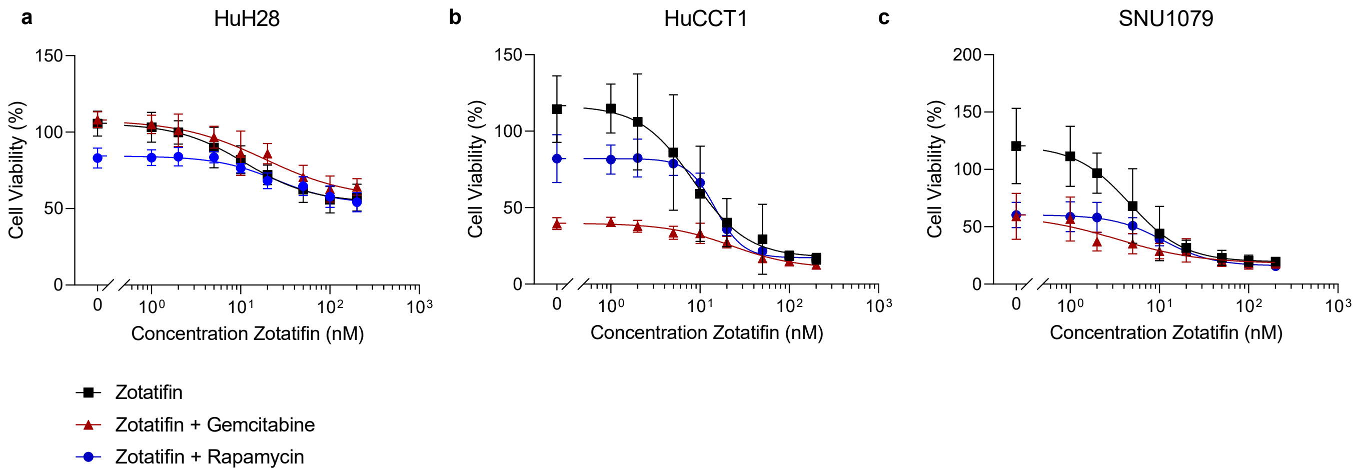

Supplementary Figure 13 – EIF4A1 inhibition in vitro.
Cell-viability titration test using eFT226 in three different cell lines. Rapamycin concentration was 200 nM and gemcitabine concentration was 1 μM for SNU-1079 and 10 μM for HuCCT-1 and HuH-28. Conditions were tested in technical quadruplicates, and experiments were independently repeated twice. Data are presented as mean ± standard deviation. Dose–response curves were fitted using a four-parameter logistic model. Source data are provided as a Source Data file.

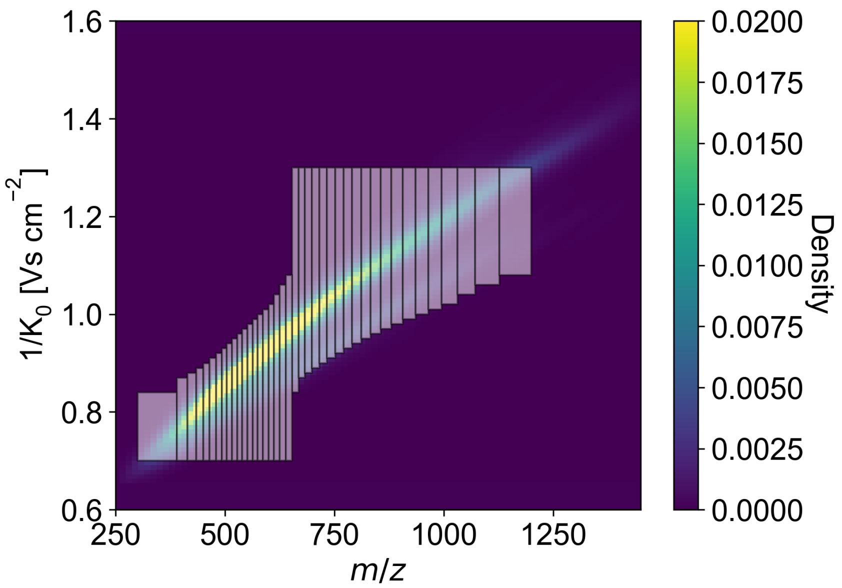

Supplementary Figure 14 – Optimized isolation windows.
Generated with pydiAID[2].

Supplementary Tables

Supplementary Table 1 - Clinical annotation of subclusters in the MSKCC-ICC cohort. PSC - Primary Sclerosing Cholangitis. HEP B/C - diagnosed Hepatitis B or C infection. Duct Type LDT – Large Duct Type; SDT – Small Duct Type.

MSKCC-ICC	N	ECM cluster N = 48[1]	Proliferation cluster N = 32[1]	p-value[2]
**Age (years)**	80	68 (61, 73)	69 (62, 74)	>0.9
**Ethnicity**	79			0.879
**A**		4 (8.5%)	4 (13%)	
**B**		2 (4.3%)	2 (6.3%)	
**H**		1 (2.1%)	0 (0%)	
**W**		40 (85%)	26 (81%)	
**Unknown**		1	0	
**Sex**	80			0.358
**F**		25 (52%)	20 (63%)	
**M**		23 (48%)	12 (38%)	
**PSC**	80	2 (4.2%)	1 (3.1%)	>0.9
**Hep B/C**	58			0.127
**N**		27 (82%)	24 (96%)	
**Y**		6 (18%)	1 (4.0%)	
**Unknown**		15	7	
**Tumor Stage**	80			0.014
**T1**		24 (50%)	7 (22%)	
**T2**		18 (38%)	21 (66%)	
**T3**		3 (6.3%)	4 (13%)	
**T4**		3 (6.3%)	0 (0%)	
**Radiotherapy**	80	12 (25%)	18 (56%)	0.005
**Duct Type**	80			0.853
**LDT**		20 (42%)	14 (44%)	
**SDT**		28 (58%)	18 (56%)	
**Lymphangioinvasion**	80	16 (33%)	24 (75%)	2.61e-04
**Satellite Nodules**	80	5 (10%)	8 (25%)	0.083
**Liver Capsule Invasion**	80	4 (8.3%)	5 (16%)	0.472
**Portal Spread**	61	4 (11%)	4 (16%)	0.706
**Unknown**		12	7	
**Overall Tumor Budding**	80			0.452
**1**		16 (33%)	7 (22%)	
**2**		18 (38%)	16 (50%)	
**3**		14 (29%)	9 (28%)	
**intratumoral Tumor Budding**	80			0.385
**1**		19 (40%)	8 (25%)	
**2**		17 (35%)	15 (47%)	
**3**		12 (25%)	9 (28%)	

**peritumoral Tumor Budding**	74			0.065
**1**		24 (53%)	9 (31%)	
**2**		17 (38%)	19 (66%)	
**3**		4 (8.9%)	1 (3.4%)	
**Unknown**		3	3	
**Liver Cells %**	74	1.14 (0.57, 2.07)	0.83 (0.41, 1.27)	0.155
**Unknown**		6	0	
**Immune Cells %**	74	3.3 (1.1, 7.6)	2.8 (0.8, 6.9)	0.347
**Unknown**		6	0	
**Necrotic Cells %**	74	0.9 (0.3, 1.9)	2.3 (0.6, 8.5)	0.005
**Unknown**		6	0	
**Stromal Cells %**	74	24 (17, 35)	17 (10, 29)	0.013
**Unknown**		6	0	
**ICC Tumor Cells %**	74	64 (52, 77)	73 (56, 81)	0.149
**Unknown**		6	0	
**[1] Median (Q1, Q3); n (%)**				
**[2] Wilcoxon rank sum test; Fisher's exact test; Pearson's Chi-squared test; Wilcoxon rank sum exact test *(all p-values are two-sided)***				

Supplementary Table 2 – Clinical annotation of assigned UKF-ICC subclusters.

UKF-ICC	N	ECM cluster N = 18[1]	Proliferation cluster N = 42[1]	p-value[2]
**Sex**	60			>0.9
**female**		10 (56%)	23 (55%)	
**male**		8 (44%)	19 (45%)	
**Age**	60	64 (59, 69)	67 (56, 73)	0.5
**Grading**	60			0.076
**G1**		4 (22%)	2 (4.8%)	
**G2**		7 (39%)	26 (62%)	
**G3**		7 (39%)	14 (33%)	
**Staging**	60			0.5
**pT1**		10 (56%)	16 (38%)	
**pT2**		3 (17%)	15 (36%)	
**pT3**		3 (17%)	8 (19%)	
**pT4**		2 (11%)	3 (7.1%)	
**Lymph Node Metastasis**	60			0.2
**pN0**		5 (28%)	19 (45%)	
**pN1**		3 (17%)	10 (24%)	
**pNx**		10 (56%)	13 (31%)	
**Lymphangiosis**	60			0.3
**L0**		14 (78%)	27 (64%)	
**L1**		4 (22%)	15 (36%)	
**Haemangiosis**	60			>0.9
**V0**		14 (78%)	31 (74%)	
**V1**		4 (22%)	11 (26%)	
**Perineural Sheath Invasion**	60			0.073
**Pn0**		9 (50%)	31 (74%)	
**Pn1**		9 (50%)	11 (26%)	
**Distant Metastasis**	60			>0.9
**M0**		15 (83%)	34 (81%)	
**M1**		2 (11%)	5 (12%)	
**NA**		1 (5.6%)	3 (7.1%)	

[1] n (%); Median (Q1, Q3)

[2] Pearson's Chi-squared test; Wilcoxon rank sum test; Fisher's exact test *(all p-values are two-sided)*

Supplementary Table 3 - Clinical annotation of assigned FU-ICC subclusters (as taken from Dong et al., 2022 [3]).

AFP – Alpha-Fetoprotein, TACE – Transarterial Chemoembolization.

FU-ICC	n	ECM cluster n = 141[1]	Proliferation cluster n = 73[1]	p-value[2]
**Sex**	214			0.2
**Female**		66 (47%)	28 (38%)	
**Male**		75 (53%)	45 (62%)	
**Age**	214	61 (54, 69)	62 (55, 70)	0.8
**Intrahepatic metastasis**	214	38 (27%)	33 (45%)	0.007
**Liver fluke**	214	11 (7.8%)	3 (4.1%)	0.4
**HBsAg (1, positive; 0, negative)**	214	36 (26%)	22 (30%)	0.5
**Biliary tract stone disease**	214	3 (2.1%)	2 (2.7%)	>0.9
**Tumor_size_diameter (cm)**	214	5.30 (3.90, 7.00)	5.80 (4.00, 8.10)	0.094
**Vascular invasion**	214	56 (40%)	41 (56%)	0.022
**Liver cirrhosis**	214	12 (8.5%)	10 (14%)	0.2
**Regional lymph Node metastasis**	214	29 (21%)	15 (21%)	>0.9
**Distal metastasis**	214	5 (3.5%)	5 (6.8%)	0.3
**Perineural invasion**	214	27 (19%)	18 (25%)	0.3
**TB: total bilirubin (μmol/L)**	214	11.0 (8.6, 15.1)	10.8 (8.1, 13.2)	0.2
**ALB, albumin, (g/L)**	214	43.0 (41.0, 47.0)	42.0 (40.0, 45.0)	0.2
**Preoperative serum AFP（ng/mL）**	214	3 (2, 5)	3 (2, 5)	0.4
**CA19-9, carbohydrate antigen 19-9 (U/mL)**	214	47 (17, 218)	44 (18, 583)	0.7
**CEA, carcinoembryonic antigen (μg/L)**	214	2 (2, 4)	3 (2, 6)	0.075
**ALT, aminoleucine transferase (U/L)**	214	20 (14, 32)	18 (13, 30)	0.5
**γ-GT, γ-glutamyltransferase (U/L)**	214	54 (24, 116)	63 (32, 104)	0.4
**TNM stage**	214			0.007
**IA**		34 (24%)	10 (14%)	
**IB**		20 (14%)	5 (6.8%)	
**II**		35 (25%)	35 (48%)	
**IIIA**		2 (1.4%)	0 (0%)	
**IIIB**		45 (32%)	18 (25%)	
**IV**		5 (3.5%)	5 (6.8%)	
**Adjuvant therapies**	214			0.7
**Chemotherapy**		33 (23%)	16 (22%)	
**No treatment**		94 (67%)	47 (64%)	
**TACE**		14 (9.9%)	10 (14%)	

[1] n (%); Median (IQR)

[2] Pearson's Chi-squared test; Wilcoxon rank sum test; Fisher's exact test *(all p-values are two-sided)*

Supplementary Table 4 - Zotatifin treatment study in mice.
Summary of Study Endpoints and Rationale for Control and Treatment Groups, n=8.

Group	Animal	Day of Euthanasia	Rationale
**Control**	1	18	Body Weight Loss
	2	24	Ulceration
	3	25	>2000% tumor volume
	4	25	>2000% tumor volume
**Treatment**	1	14	Ulceration
	2	28	>2000% tumor volume
	3	35	>2000% tumor volume
	4	35	End of Study

Supplementary Methods

Ethics Statement

ICC tumors for this study were collected in the Memorial Sloan Kettering Cancer Center New York (MSKCC), USA, between 2009 and 2018. The MSKCC institutional review board approved the study (protocol number 16-1683A(3)). A second cohort of ICC tumors was collected at the Medical Center - University of Freiburg (UKF), Germany, between 2000 and 2022 with approval of the Institutional Ethics Committee (protocol number 21-1684). Written informed consent was obtained from patients of both MSKCC and UKF before inclusion. The studies were conducted according to the Declaration of Helsinki. Mouse xenograft experiments for generation of FFPE samples were approved by the Committee on the Ethics of Animal Experiments of the regional council (Permit Number I-19/02). Mouse experiments for the treatment study were approved by the German Committee on the Ethics of Animal Experiments of the regional council (Permit Number G-20/163). The mouse study was carried out in strict accordance with the recommendations in the Guide for the Care and Use of Laboratory Animals of the Society of Laboratory Animals (GV SOLAS) in an AAALAC accredited animal facility.

ICC Cohorts

All tissue specimens originating from the MSKCC were formalin-fixed, paraffin-embedded (FFPE), and sectioned into 10 µm slices at the MSKCC Department of Pathology. All histological slides were evaluated by two experienced pathologists at MSKCC according to the World Health Organization classification [4]. Tumor staging was conducted according to the 8th edition of the TNM classification system by the American Joint Committee on Cancer (AJCC) [5]. Corresponding clinical data encompassed patient demographics (age, sex, ethnicity), infection status (hepatitis B/C) and tumor characteristics (staging, resection date, first recurrence data, last follow-up date, and tumor budding [determined by Budau et al. [6]]). For hematoxylin and eosin (HE)-stained tissue classification and automated quantification of tumor cell percentage, we trained a classifier ("Object Phenotyper") using the HALO AI module (version 4.0.5107) on the HALO Image Analysis Platform (version 4.0.5107, Indica Labs, Inc.). Nuclei segmentation was performed with the *Nuclei Seg – BF v1.0.0* network within HALO AI. The classifier was trained in a supervised manner with more than 150,000 manually annotated nuclei by a surgical pathology resident. Annotated cell categories included lymphocytes, hepatocytes, necrotic cells, stromal cells, and viable tumor cells. Training was stopped after approximately 14,000 iterations, reaching a cross-entropy of 0.008. Classifier performance was assessed qualitatively by visual inspection of each quantification output through a trained pathologist to verify accurate cell type classification and tumor cell percentage estimation.

Tissue specimens from the UKF were also formalin-fixed and paraffin-embedded. From each FFPE block, 1 mm cores of tumor and tumor-adjacent tissue were punched at the UKF Institute for Surgical Pathology and provided by the Biobank Comprehensive Cancer Center Freiburg. All histological samples were reviewed by experienced pathologists. Tumors were staged according to the AJCC TNM classification system [5]. Additional clinical data included patient demographics (age, sex), and tumor characteristics (staging, grading, lymphovascular, vascular and perineural sheath invasion, residual disease status), and survival outcomes.

Sample Preparation for LC-MS/MS

All tissue slides from the MSKCC cohort underwent deparaffinization in consecutive baths in xylol, 99%, 96%, 70%, and 50% ethanol. Slides were stored in water until the tumor and tumor-adjacent, non-malignant (TANM) tissue were macrodissected by a pathologist. Each sample was then separately transferred into reaction tubes and stored at -80°C. For protein extraction, samples were suspended in 0.1% RapiGest (Waters, Milford, MA, USA) and 100 mM 4-(2-hydroxyethyl)-1-piperazineethanesulfonic acid (HEPES), sonicated with a BioRuptor (Diagenode, Seraing, Belgium) in 20 cycles à 30 s and heated at 90 °C for 1.5 h [7]. Resulting protein concentrations were quantified via a bicinchoninic acid assay (BCA; ThermoFisher Scientific, Walldorf, Germany) and up to 100 µg of protein per sample were reduced with 5 mM dithiothreitol (Sigma Aldrich, Steinheim, Germany) and incubated in the dark for 30 min. After subsequent alkylation with 10 mM iodoacetamide (Sigma Aldrich) and incubation at 37 °C for 30 min, samples were digested with 1:50 (w/w) lysyl endopeptidase C (Fujifilm, Santa Ana, CA, USA) at 42 °C for 2 h and 1:50 (w/w) trypsin (Worthington Biochemicals, Lakewood, NJ, USA) at 37 °C overnight. RapiGest was hydrolyzed and removed by the addition of 2% trifluoroacetic acid, incubation at 37 °C for 30 min, and 15 min centrifugation at 21,000 x g. The peptide containing supernatant was desalted using a solid phase peptide extraction kit (PreOmics, Planegg, Germany) according to the manufacturer's instructions. Peptide concentrations were again determined via BCA and 2 µg of peptides per sample were aliquoted for LC-MS/MS measurement and vacuum dried after the addition of 200 fmol of indexed retention time standards (iRTs; Biognosys, Schlieren, Switzerland).

FFPE cores from the UKF cohort were manually deparaffinized in sequential xylene and ethanol washes (100%, 96%, 70%, 50%) and dried in a vacuum concentrator. For protein extraction, samples were suspended in 100 µl 100 mM HEPES (pH 8.0), homogenized using a PreCellys device (Bertin GmbH, Frankfurt am Main, Germany), and supplemented with Sodium dodecyl sulfate (SDS) to 1% (v/v). Antigen retrieval was performed at 95 °C for 1 h with shaking, followed by ultrasonication in a BioRuptor (20 cycles, 40 s on/20 s off, high intensity) and a second heating step at 95 °C for 30 min. Lysates were cleared by centrifugation (21,300 × g, 5 min) and protein concentrations were determined by BCA assay. Up to 20 µg protein in the extraction buffer (100 mM HEPES, 1% SDS) were transferred to 96-well plates for automated processing on an automated liquid handling platform (Bravo, Agilent Technologies, Santa Clara, CA, USA). Sample preparation followed a single-pot, solid-phase-enhanced sample preparation (SP3) protocol [8]. Proteins were reduced with 5 mM Tris (2-carboxyethyl)phosphine hydrochloride (TCEP) (Sigma-Aldrich) and alkylated with 20 mM 2-Chloroacetamide (CAA) (Sigma-Aldrich; 30 min, 37 °C). Proteins were bound to washed SP3 beads (Cytiva, Marlborough, MA, USA) in 50% acetonitrile, washed with 70% ethanol and 100% acetonitrile, and resuspended in 100 mM ammonium bicarbonate. Digestion was performed with lysyl endopeptidase C (Serva, Heidelberg, Germany; 1:50, w/w, 2 h, 42 °C) and trypsin (Promega, Madison, WI, USA; 1:25, w/w, overnight, 37 °C). Digestion was stopped with 1% trifluoroacetic acid, and peptides were collected after bead removal. Peptide concentrations were determined by BCA, and 2 µg peptide per sample were spiked with 400 fmol iRT standards. Samples were vacuum dried at 45 °C and stored at –80 °C until LC-MS/MS measurement.

Patient-derived Xenograft Proteomics

FFPE tumor sections from nine different patient-derived ICC xenograft (PDX) mouse models were provided by Charles River Laboratories Germany (Freiburg). Patient tissues did not originate from the MSKCC-ICC or UKF-ICC cohorts but were collected independently. Tumor tissue was propagated as described before [9]. The study was carried out in accordance with the recommendations of GV-SOLAS in an AAALAC-accredited animal facility. All animal experiments were approved by the Committee on the Ethics of Animal Experiments of the regional council (Permit Numbers I-19/02). ICC PDX models were implanted subcutaneously in 4-6-week-old female NMRI nu/nu mice (Charles River Laboratories, Sulzfeld, Germany) under isoflurane anesthesia. Tumor growth was determined by a two-dimensional measurement with calipers twice a week. Analysis of the PDX models were performed when tumor size reached 800–1000 mm^3. Tumors were sampled, fixed for 24 h in formalin, and subsequently embedded in paraffin. Deparaffinization was performed as described above. 4% SDS in 0.1 M HEPES was added to the tissue, which was then sonicated with a BioRuptor in 20 cycles à 40 s and boiled at 95 °C for 90 min. Reduction and alkylation were performed by adding 5 mM TCEP and 10 mM CAA with subsequent incubation for 30 min at 37 °C in the dark. After centrifugation, 12% aqueous phosphoric acid was added to the sample supernatant in a 1:10 ratio. Upon addition of a 3-fold excess volume of 90% methanol containing 0.1 M triethylammonium bicarbonate (TEAB), the sample was loaded onto an S-Trap micro column (ProTifi, Fairport, NY, USA) and then washed three times with the same buffer. Trypsin was added in a ratio of 1:10 (w/w) in a 50 mM TEAB buffer and incubated for 2 h at 47 °C. Afterwards, digested peptides were eluted in three steps: first in 50 mM TEAB, second in 0.2% formic acid, and third in 50% acetonitrile containing 0.2% formic acid. Subsequently, samples were aliquoted as described above and a library was generated with input from all PDX samples.

LC-MS/MS Measurement

Peptides from the MSKCC cohort (n = 86 tumors and 69 TANM samples) were measured on a Bruker TimsTOF Flex mass spectrometer (Bruker, Bremen, Germany) coupled to an Evosep One chromatography system (Evosep, Odense, Denmark). 1 µg dried peptides were dissolved in 40 µl aqueous buffer A containing 0.1% formic acid, sonicated for 5 min, and centrifuged for 5 min at 21,000 x g. 20 µl, corresponding to 500 ng of peptides, were loaded on prewashed and preconditioned Evotips (Evosep) according to the manufacturer's protocol. Chromatographic separation was performed with the vendor's standard 30 samples per day (30 SPD) method using a 44 min gradient at 500 nl/min and an EV 1137 performance 15 cm C18 reverse-phase column. Mobile phases A and B consisted of 0.1% v/v formic acid in ddH_2O and 0.1% v/v formic acid in acetonitrile, respectively. Measurements were performed in data-independent acquisition (DIA) mode, combined with parallel accumulation-serial fragmentation (dia-PASEF). MS scans covered an m/z range of 100-1700 m/z and an ion mobility range of $1/K_0$ = 0.70–1.30 V·s cm^{-2}. Accumulation and ramp time were both set to 100 ms, corresponding to a 100% duty cycle. The DIA window scheme was optimized using pydiAID [2]. In brief, a previously generated pool of peptides from all samples was measured in technical triplicates. To optimize fragmentation efficiency, the resulting data was used to adjust ion mobility and isolation windows to contain equal amounts of precursors. These custom windows were then used for all measurements of the MSKCC-ICC cohort. The final method comprised 20 MS/MS scans, each with two ion mobility windows, covering a range of 300–1200 m/z across the defined mobility range (Supplementary Fig. 14). The total cycle time was 2.23 s.

UKF (n = 66 patient-matched tumor and TANM samples) and PDX samples (n = 9) were measured on a Q Exactive Plus mass spectrometer coupled to an Easy-nLC 1000 nano-flow HPLC (both TermoFisher Scientific, Waltham, MA, USA). Dried peptides were dissolved in 10 µl ddH_2O, sonicated for 5 min and centrifuged for 10 min at 21,300 x g. The supernatant was transferred into vials and 4 µl, equivalent to 800 ng of peptides, were injected for measurement. The column setup of the LC device consisted of an Acclaim™ PepMap™ 100 C18 column and a 200 cm µPac C18 nano-LC analytical column (both ThermoFisher Scientific) coupled to a Nanospray FlexTM ion source and a fused silica emitter. Peptides were separated over a 120 min gradient with buffer A (0.1% formic acid in water) and buffer B (0.1% formic acid in 80% acetonitrile) as follows: 8–10% B in 2 min, 10–20% B in 22 min, 20–40% B in 46 min, 40–55% B in 10 min, and 55–100% B in 2 min. Samples were measured in DIA mode in a scan range from 385 to 1015 m/z in staggered acquisition windows of 24 m/z each [10,11]. All measurements were performed with a resolution of 17,500 and a maximum injection time of 80 ms. Peptides were fragmented via Higher-energy C-trap dissociation (HCD) at stepped normalized collision energies of 25 and 30.

No measurement replicates were performed due to limited material availability.

DNA extraction and WES

DNA of tumor and TANM samples was extracted using the QIAamp DNA FFPE Tissue Kit (Qiagen, Hilden, Germany) according to the manufacturer's protocol. DNA concentration was measured on a Tape Station (Agilent) and quality was checked on a bioanalyser. WES libraries were prepared with Novogene's library preparation kit followed by exon capture with the Agilent v6 kit. Sequencing was performed on the Illumina NovaSeq X Plus platform (Illumina, San Diego, CA, USA) with PE150 sequencing strategy generating ~12 Gb (mean coverage: 208X, range: 55-375X) of raw data for tumor samples and 24 Gb (mean coverage: 109X, range: 20-213X) for TANM samples.

Cell Culture

Three ICC cell lines were used for cell culture experiments. HuCC-T1 (#JCRB0425) and HuH-28 (#JCRB0426) cell lines were obtained from the Japanese Collection of Research Bioresources Cell Banks (JCRB), and SNU-1079 (#CSC-C9622L) cells were obtained from the Korean Cell Line Bank (KCLB). Cells were cultured in Roswell Park Memorial Institute (RPMI) 1640 medium supplemented with 10% fetal bovine serum (FBS, PAN Biotech, Aidenbach, Germany) and 1x penicillin/streptomycin (Gibco, Thermo Fisher Scientific, New York, NY, USA) at 37 °C in a 5% CO_2 humidified atmosphere. Cell line authentication was verified by Eurofins Genomics, DNA isolation was carried out from cell pellets and genetic characteristics were determined by PCR-single-locus-technology. All cell lines were tested negative for mycoplasma contamination by Eurofins.

For viability assays, cells were seeded at a density of 3,000 cells/well (HuCC-T1 and SNU-1079) or 6,000 cells/well (HuH-28) in 96-well flat-bottom plates in 90 µl of growth medium and cultured overnight. Cells were treated with different concentrations of eFT226 (Zotatifin; MedChemExpress, Monmouth Junction, NJ, USA; solubilized in DMSO) as a single agent or in combination with either gemcitabine (Sigma-Aldrich; solubilized in H_2O) or rapamycin (Sigma-Aldrich; dissolved in DMSO). For the gemcitabine-high-sensitive SNU-1079 cell line, a concentration of 1 µM was used, while a concentration of 10 µM was used for the other cell

lines. Rapamycin was applied at 200 nM for all cell lines. The final DMSO concentration was ~0.1%. Cells were incubated for 48 h at 37 °C in a 5% CO_2 atmosphere. DMSO-treated cells and wells with only culture medium served as negative and background controls, respectively. Cell viability was measured using the MTT assay (CellTiter 96® Non-Radioactive Cell Proliferation Assay, Promega, Madison, WI, USA) following the manufacturer's protocol. Briefly, 15 µl of MTT dye reagent was added and incubated for 4 h at 37 °C. Subsequently, 100 µl of stop solution was added, and the plate was further incubated for 1 h at 37 °C and 500 rpm in a thermoshaker. The average absorbance of DMSO-treated cells was set as 100% cell viability. All conditions were tested in technical quadruplicates, and experiments were independently repeated twice. Viability curves were generated with GraphPad Prism version 8.3.0 using a 4-parameter model.

PDX Treatment Study

This study was carried out in strict accordance with the recommendations in the Guide for the Care and Use of Laboratory Animals of the Society of Laboratory Animals (GV SOLAS). All animal experiments were approved by the Committee on the Ethics of Animal Experiments of the regional council (Permit Numbers: G-20/163). Four- to six-week-old female $NMRI^{nu/nu}$ mice (Charles River, Germany) placed under isoflurane anesthesia received tumor implants subcutaneously in one flank. Animals were housed in individually ventilated cages (TECNIPLAST Sealsafe-IVC System, TECNIPLAST, Hohenpeissenberg, Germany), depending on group size, either in type III or type II long cages. They were kept under a 14L:10D artificial light cycle. All interventions were done during light cycle. Temperature inside the cages was maintained at 22–26 °C with a relative humidity of 45–65% and 60–65 air changes/hour in the cage. Dust-free bedding consisting of aspen wood chips with approximate dimensions of 5 mm × 5 mm × 1 mm (ABEDD, LAB & VET Service GmbH, Vienna, Austria, product code: LTE E 001) and additional nesting material were used. The cages including the bedding and nesting material were changed weekly. Animals were fed a breeding diet for rats and mice (Altromin 1314 P fortified, irradiated) and had access to sterile filtered and acidified (pH 2.5) tap water that was changed twice weekly. Feed and water were provided *ad libitum*. All materials were autoclaved prior to use. No fasting was done at any time point of the study.

Animals and tumor implants were monitored daily until the maximum number of implants showed clear signs of beginning solid tumor growth. Tumor growth was determined by a two-dimensional measurement with calipers biweekly together with body weight determination. Tumor volumes were calculated according to the following equation:

Tumor Vol [mm^3] = a [mm] x b^2 [mm^2] x 0.5, where a is the largest diameter and b is the perpendicular diameter of the tumor representing an idealized ellipsoid. Animals bearing 75–240 mm^3 tumors were distributed into experimental groups, with comparable median and mean tumor volumes. The day of randomization was designated as day 0 of an experiment and dosing was started in the next 24 h. Test compound eFT226 was administered intravenously at 1 mg/kg/day once weekly for three weeks. The vehicle, 5% dextrose in water for injection, was administered at the same route and schedule. The relative volume of an individual tumor on day X (RTV_x) was calculated by dividing the absolute volume [mm^3] of the respective tumor on day X (T_x) by the absolute volume of the same tumor on the day of randomization, i. e. on day 0 (T_0), multiplied by 100, as shown by the following equation:

$$RTV_x\ [\%] = T_x/T_0 \times 100$$

Tumor inhibition on a particular day (T/C_x) was calculated from the median RTV of a test group and the median RTV of a control group multiplied by 100, as shown by the following equation:

$$T/C_x \text{ [\%]} = \text{median } RTV_x \text{ treated group/ median } RTV_x \text{ control group} \times 100$$

The minimum T/C [%] value recorded for a particular group during an experiment represented the maximum anti-tumor efficacy for the respective compound. T/C values were only considered valid if at least 50% of the randomized animals in the test and in the control group were alive on the day in question. Moderate efficacy was defined as 25% ≤ T/C ≤ 50%.
The maximal permitted tumor burden was a tumor volume of 2,000 mm^3. Animals were monitored regularly and were sacrificed when tumor volume reached or exceeded this limit. In addition, Last Observation Carried Forward (LOCF) data were included in the calculation if applicable. In the LOCF methodology, tumor volumes of animals that were euthanized due to their tumor load are carried forward for as long as this increases the group median tumor volume.

Proteomic Data Analysis

Library generation and peptide-to-spectrum matching for patient cohort samples were performed in DIA-NN version 1.9.2 [12] by using a human proteome datafile containing reviewed sequences as downloaded from Uniprot on May 7th, 2024. A predicted library was generated, with Trypsin/P specificity, allowing one missed cleavage and with N-term M excision and C carbamidomethylation. Peptide length range was set to 7–30 amino acids and false discovery rate (FDR) to 1%. The same settings were applied for peptide-to-spectrum matching with match between runs (MBR) enabled. Protein quantification was performed via the MaxQuant label-free quantification (LFQ) algorithm as implemented in DIA-NN. To analyze semi-specific peptides, library files were refined against a human proteome database containing all possible semi-specific peptide sequences. 1% FDR was applied and no missed cleavage sites were permitted for library generation and reannotation of peptides of 7–30 amino acids length.

All subsequent data analysis was performed in R (version 4.4.2) using RStudio (version 2026.01.0) and in-house scripts [13,14]. The DIA-NN output was exported as an expression matrix by using the DIA-NN R package (version 1.0.1) [15] and then log2-transformed and median-normalized. Hierarchical clustering and principal component analysis (PCA) were performed with MixOmics (version 6.20.0), Monte-Carlo simulation with M3C (version 1.18) [16,17]. For linear modeling, we used the limma package (version 3.52.4) [18] with a fixed-effects design comparing tumor versus TANM, without random intercepts for individual patients. Potential confounding variables were not included in the model. FDR was controlled using the Benjamini-Hochberg method. Network analyses were performed using Cytoscape (version 3.10.0) [19] and enrichment analyses via ClusterProfiler (version 4.9.0) [20] using KEGG [21,22], Reactome [23], and Gene Ontology [24,25] databases. Survival statistics, including Cox proportional hazards model, were applied via the survival (version 3.4-0) [26] and survminer (version 0.4.9) [27] packages.

The Cox proportional hazards model was fitted to expression values of each protein iteratively. Patients who did not experience a recurrence or death throughout the study were censored at the last follow-up and included in the analysis. Radiotherapy was included as a covariate, as it was the only independent clinical parameter significantly associated with survival. The resulting list of protein candidates was adjusted for multiple-testing errors via Benjamini-Hochberg FDR correction.

To transfer cluster identities from our cohort to the external cohort published by Dong et al. [3], and to our UKF-ICC cohort, median-centered datasets were fused via sva (version 3.44.0) [28,29] by designating each cohort as one batch. Using the xgboost package (v. 1.7.8.1) [30], a classifier was iteratively trained, tested, and optimized on the MSKCC-ICC part of the fused datasets. The resulting classifiers were then applied to the FU-ICC cohort and the UKF-ICC cohort.

Analysis of proteolytic processing was performed using in-house-developed R scripts for the annotation and visualization of semi-specific peptides (publicly available on GitHub as the TermineR package version 1.0.0) [31].

Xenograft samples were analyzed via DIA-NN 1.7, the DIA-NN R package and MixOmics as described above [11,14,15]. Here, a combined human-mouse proteome datafile as downloaded from Uniprot on September 1st, 2020 was used for reannotation.

WES Data Analysis

Cleaned reads were mapped to the GRCh38/hg38 p13 reference genome using Burrows-Wheeler Aligner (BWA; version 0.7.17), and resulting BAM files were processed with Sambamba (version 1.0.0) and Picard (version 2.18.9) [32–34]. Germline variants were called with GATK (version 4.3.0), somatic variants were detected by MuTect (version 2.2-25-g268eab) and Strelka (version 2.9.10) and annotated using ANNOVAR [35–38]. SNPs were filtered for a minimum coverage of 10 reads, variant allele fraction (VAF) > 5%, and ≥ 5 supporting reads in the tumor. Indels were filtered for VAF >10%. Tumor mutational burden (TMB) was calculated as the number of non-synonymous somatic mutations (after filtering) divided by the total size of the target coding regions.

Supplementary References

1. Luo, W. & Brouwer, C. Pathview: an R/Bioconductor package for pathway-based data integration and visualization. *Bioinformatics* **29**, 1830–1831 (2013).
2. Skowronek, P. *et al.* Rapid and In-Depth Coverage of the (Phospho-)Proteome With Deep Libraries and Optimal Window Design for dia-PASEF. *Molecular & Cellular Proteomics* **21**, (2022).
3. Dong, L. *et al.* Proteogenomic characterization identifies clinically relevant subgroups of intrahepatic cholangiocarcinoma. *Cancer Cell* **40**, 70-87.e15 (2022).
4. Bosman, F. T., Carneiro, F., Hruban, R. H. & Theise, N. *WHO Classification of Tumours of the Digestive System*. (2010).
5. AJCC Cancer Staging Manual. https://link.springer.com/book/9783319406176.
6. Budau, K.-L. *et al.* Prognostic Impact of Tumor Budding in Intrahepatic Cholangiocellular Carcinoma. *J Cancer* **13**, 2457–2471 (2022).
7. Föll, M. C. *et al.* Reproducible proteomics sample preparation for single FFPE tissue slices using acid-labile surfactant and direct trypsinization. *Clin Proteomics* **15**, 11 (2018).
8. Müller, T. *et al.* Automated sample preparation with SP3 for low-input clinical proteomics. *Mol Syst Biol* **16**, e9111 (2020).
9. Fiebig, H.-H. *et al.* Gene signatures developed from patient tumor explants grown in nude mice to predict tumor response to 11 cytotoxic drugs. *Cancer Genomics Proteomics* **4**, 197–209 (2007).
10. Ludwig, C. *et al.* Data-independent acquisition-based SWATH-MS for quantitative proteomics: a tutorial. *Mol Syst Biol* **14**, e8126 (2018).
11. Vidova, V. & Spacil, Z. A review on mass spectrometry-based quantitative proteomics: Targeted and data independent acquisition. *Anal Chim Acta* **964**, 7–23 (2017).
12. Demichev, V., Messner, C. B., Vernardis, S. I., Lilley, K. S. & Ralser, M. DIA-NN: neural networks and interference correction enable deep proteome coverage in high throughput. *Nat Methods* **17**, 41–44 (2020).
13. R Core Team. R: A Language and Environment for Statistical Computing. R Foundation for Statistical Computing, Vienna, Austria (2023).
14. Posit team. RStudio: Integrated Development Environment for R. Posit Software, PBC, Boston, MA (2025).
15. Demichev, V. diann: Report processing and protein quantification for MS-based proteomics. (2020).
16. Rohart, F., Gautier, B., Singh, A. & Cao, K.-A. L. mixOmics: An R package for 'omics feature selection and multiple data integration. *PLOS Computational Biology* **13**, e1005752 (2017).
17. John, C. R. *et al.* M3C: Monte Carlo reference-based consensus clustering. *Sci Rep* **10**, 1816 (2020).
18. Smyth, G. K. limma: Linear Models for Microarray Data. in *Bioinformatics and Computational Biology Solutions Using R and Bioconductor* (eds. Gentleman, R., Carey, V. J., Huber, W., Irizarry, R. A. & Dudoit, S.) 397–420 (Springer-Verlag, New York, 2005). doi:10.1007/0-387-29362-0_23.
19. Shannon, P. *et al.* Cytoscape: a software environment for integrated models of biomolecular interaction networks. *Genome Res* **13**, 2498–2504 (2003).
20. Wu, T. *et al.* clusterProfiler 4.0: A universal enrichment tool for interpreting omics data. *Innovation (Camb)* **2**, 100141 (2021).
21. Kanehisa, M. & Goto, S. KEGG: kyoto encyclopedia of genes and genomes. *Nucleic Acids Res* **28**, 27–30 (2000).

22. Kanehisa, M., Furumichi, M., Sato, Y., Kawashima, M. & Ishiguro-Watanabe, M. KEGG for taxonomy-based analysis of pathways and genomes. *Nucleic Acids Res* **51**, D587–D592 (2023).
23. Gillespie, M. *et al.* The reactome pathway knowledgebase 2022. *Nucleic Acids Res* **50**, D687–D692 (2022).
24. Ashburner, M. *et al.* Gene Ontology: tool for the unification of biology. *Nat Genet* **25**, 25–29 (2000).
25. Gene Ontology resource: enriching a GOld mine | Nucleic Acids Research | Oxford Academic. https://academic.oup.com/nar/article/49/D1/D325/6027811.
26. Therneau, T. M. A Package for Survival Analysis in R. (2020).
27. Kassambara, A., Kosinski, M. & Biecek, P. survminer: Drawing Survival Curves using 'ggplot2'.
28. Johnson, W. E., Li, C. & Rabinovic, A. Adjusting batch effects in microarray expression data using empirical Bayes methods. *Biostatistics* **8**, 118–127 (2007).
29. Nyamundanda, G., Poudel, P., Patil, Y. & Sadanandam, A. A Novel Statistical Method to Diagnose, Quantify and Correct Batch Effects in Genomic Studies. *Sci Rep* **7**, 10849 (2017).
30. Chen, T. & Guestrin, C. XGBoost: A Scalable Tree Boosting System. in *Proceedings of the 22nd ACM SIGKDD International Conference on Knowledge Discovery and Data Mining* 785–794 (2016). doi:10.1145/2939672.2939785.
31. Cosenza-Contreras, M., Huesgen, P. F. & Schilling, O. TermineR: Bioinformatic processing of shotgun proteomics data for the annotation and quantitation of protein termini. *Methods Enzymol* **719**, 43–66 (2025).
32. Li, H. & Durbin, R. Fast and accurate long-read alignment with Burrows-Wheeler transform. *Bioinformatics* **26**, 589–595 (2010).
33. Tarasov, A., Vilella, A. J., Cuppen, E., Nijman, I. J. & Prins, P. Sambamba: fast processing of NGS alignment formats. *Bioinformatics* **31**, 2032–2034 (2015).
34. Picard Tools - By Broad Institute. https://broadinstitute.github.io/picard/.
35. DePristo, M. A. *et al.* A framework for variation discovery and genotyping using next-generation DNA sequencing data. *Nat Genet* **43**, 491–498 (2011).
36. Cibulskis, K. *et al.* Sensitive detection of somatic point mutations in impure and heterogeneous cancer samples. *Nat Biotechnol* **31**, 213–219 (2013).
37. Saunders, C. T. *et al.* Strelka: accurate somatic small-variant calling from sequenced tumor-normal sample pairs. *Bioinformatics* **28**, 1811–1817 (2012).
38. Wang, K., Li, M. & Hakonarson, H. ANNOVAR: functional annotation of genetic variants from high-throughput sequencing data. *Nucleic Acids Research* **38**, e164–e164 (2010).
