# Supplementary material for: Proteomic characterization of intrahepatic cholangiocarcinoma identifies risk-stratifying subgroups and EIF4A1 as a therapeutic target
Source: Nat Commun. 2026 Mar 23;17:2741. doi: 10.1038/s41467-026-70817-1 (PMC13013968; doi:10.1038/s41467-026-70817-1)
Supplement: Supplementary file 2 — Description of Additional Supplementary Files [file 41467_2026_70817_MOESM2_ESM.pdf]

Description of Additional Supplementary Files

File Name: Supplementary Data 1 – Patient Table MSKCC-ICC
Description: Clinical and pathological information for all patients included in the MSKCC-ICC cohort.

File Name: Supplementary Data 2 – Protein Expression Matrix MSKCC-ICC
Description: Log2 transformed and median-normalized protein intensity matrix for the MSKCC-ICC cohort including tumor and TANM samples.

File Name: Supplementary Data 3 – Differential Proteins MSKCC-ICC Tumor vs. TANM and ECM vs. Proliferation cluster
Description: Results of differential abundance analysis (limma) MSKCC-ICC cohort comparing tumor vs. TANM samples and ECM vs. Proliferation clusters, including fold changes, and adjusted p-values.

File Name: Supplementary Data 4 – Cox Proportional Hazards Model MSKCC
Description: Results of the Cox proportional hazards analysis for the MSKCC-ICC cohort showing proteins significantly associated with time to recurrence and overall survival and corresponding statistics.

File Name: Supplementary Data 5 – Protein Expression Matrix UKF-ICC
Description: Log2 transformed and median-normalized protein intensity matrix for the UKF-ICC cohort.

File Name: Supplementary Data 6 – Mutation List UKF-ICC
Description: Somatic mutation data for the UKF-ICC cohort obtained from whole-exome sequencing, including gene annotations and variant classifications.

File Name: Supplementary Data 7 – Protein Expression Matrix PDX
Description: Log2 transformed and median-normalized protein intensity matrix for patient-derived xenograft (PDX) samples.

File Name: Supplementary Data 8 – eFT226 PDX Study
Description: Data from the eFT226 PDX treatment study, including tumor volumes, body weight and statistics.

File Name: Supplementary Data 9 – Patient Table UKF-ICC
Description: Clinical and pathological information for all patients included in the UKF-ICC cohort.
