# Supplementary material for: Proteomic characterization of intrahepatic cholangiocarcinoma identifies risk-stratifying subgroups and EIF4A1 as a therapeutic target
Source: Nat Commun. 2026 Mar 23;17:2741. doi: 10.1038/s41467-026-70817-1 (PMC13013968; doi:10.1038/s41467-026-70817-1)
Supplement: Supplementary file 12 — Reporting Summary [file 41467_2026_70817_MOESM12_ESM.pdf]

nature portfolio

Corresponding author(s): Oliver Schilling

Last updated by author(s): Jan 23, 2026

Reporting Summary

Nature Portfolio wishes to improve the reproducibility of the work that we publish. This form provides structure for consistency and transparency in reporting. For further information on Nature Portfolio policies, see our Editorial Policies and the Editorial Policy Checklist.

Statistics

For all statistical analyses, confirm that the following items are present in the figure legend, table legend, main text, or Methods section.

n/a	Confirmed	
☐	☒	The exact sample size (n) for each experimental group/condition, given as a discrete number and unit of measurement
☐	☒	A statement on whether measurements were taken from distinct samples or whether the same sample was measured repeatedly
☐	☒	The statistical test(s) used AND whether they are one- or two-sided<br>*Only common tests should be described solely by name; describe more complex techniques in the Methods section.*
☐	☒	A description of all covariates tested
☐	☒	A description of any assumptions or corrections, such as tests of normality and adjustment for multiple comparisons
☐	☒	A full description of the statistical parameters including central tendency (e.g. means) or other basic estimates (e.g. regression coefficient) AND variation (e.g. standard deviation) or associated estimates of uncertainty (e.g. confidence intervals)
☐	☒	For null hypothesis testing, the test statistic (e.g. F, t, r) with confidence intervals, effect sizes, degrees of freedom and P value noted<br>*Give P values as exact values whenever suitable.*
☒	☐	For Bayesian analysis, information on the choice of priors and Markov chain Monte Carlo settings
☒	☐	For hierarchical and complex designs, identification of the appropriate level for tests and full reporting of outcomes
☐	☒	Estimates of effect sizes (e.g. Cohen's d, Pearson's r), indicating how they were calculated

Our web collection on statistics for biologists contains articles on many of the points above.

Software and code

Policy information about availability of computer code

Data collection	Peptides from the MSKCC cohort were analyzed on a Bruker timsTOF Flex mass spectrometer (Bruker) coupled to an Evosep One system (Evosep) . Peptides were loaded on Evotips and separated using the 30 samples per day method (44 min gradient, 500 nl/min) on a 15 cm C18 column. Data were acquired in DIA mode combined with dia-PASEF (m/z 100–1700, ion mobility 1/K_0 = 0.70–1.30 V·s cm^{-2}; accumulation and ramp time 100 ms). DIA windows were optimized using pydiAID, yielding 20 MS/MS scans with two ion mobility windows per scan, covering 300–1200 m/z (cycle time = 2.23 s).UKF and PDX cohort peptides were analyzed on a Q Exactive Plus mass spectrometer coupled to an EasynLC 1000 (Thermo Fisher Scientific). Peptides (800 ng) were separated over a 120 min gradient (8–100 % buffer B) and measured in DIA mode (scan range 385–1015 m/z, 24 m/z staggered windows, resolution 17,500, max injection time 80 ms, HCD collision energies 25 and 30). Libraries for Whole Exome Sequencing were prepared with Novogene's kit and Agilent v6 exon capture, sequenced on an Illumina NovaSeq X Plus (PE150), generating ~12 Gb for tumors (mean 208×) and 24 Gb for TANM samples (mean 109×).
Data analysis	All proteomic data analysis was performed in R using RStudio and in-house scripts. The DIA-NN output was exported as an expression matrix by using the DIA-NN R package (v. 1.0.1) and then log2 transformed and median-normalized. Hierarchical clustering and principal component analysis (PCA) were performed with MixOmics (v. 6.20.0), Monte-Carlo simulation with M3C (v. 1.18). For linear modeling we used the Limma package (v. 3.52.4). Enrichment analyses were performed via ClusterProfiler (v. 4.9.0) using KEGG, Reactome, and GeneOntology databases. Survival statistics, including Cox proportional hazards model, were applied via the survival (v. 3.4-0) and survminer (v. 0.4.9) packages.<br>To transfer cluster identities from our cohort to the external cohort published by Dong et al., both median-centered datasets were fused via sva (v. 3.44.0) by designating each cohort as one batch. Using the xgboost package (v. 1.7.8.1), a classifier was iteratively trained, tested, and optimized on the MSKCC-ICC part of the fused dataset. The resulting classifier was then applied to the FU-ICC cohort.<br>Analysis of proteolytic processing was performed using in-house-developed R scripts for the annotation and visualization of semi-specific peptides (publicly available on GitHub as the TermineR package v. 1.0.0).<br>Xenograft samples were analyzed via DIA-NN 1.7, the DIA-NN R package (v. 1.0.1) and MixOmics as described above.

For WES data analysis, cleaned reads were mapped to the hg38 p13 reference genome using BWA (v0.7.17), and resulting BAM files were processed with Sambamba (v1.0.0) and Picard (v2.18.9). Germline variants were called with GATK (v4.3.0), somatic variants were detected by MuTect (v2.2-25-g268eab) and Strelka (v2.9.10) and annotated using ANNOVAR . SNPs were filtered for a minimum coverage of 10 reads, variant allele fraction (VAF) >5%, and ≥5 supporting reads in the tumor. Indels were filtered for VAF >10%.
All code used to analyze data and generate figures and tables is available via Github (https://github.com/SchillingLabProteomics/ICC/tree/main#).

For manuscripts utilizing custom algorithms or software that are central to the research but not yet described in published literature, software must be made available to editors and reviewers. We strongly encourage code deposition in a community repository (e.g. GitHub). See the Nature Portfolio guidelines for submitting code & software for further information.

Data

Policy information about availability of data

All manuscripts must include a data availability statement. This statement should provide the following information, where applicable:

- Accession codes, unique identifiers, or web links for publicly available datasets
- A description of any restrictions on data availability
- For clinical datasets or third party data, please ensure that the statement adheres to our policy

All mass spectrometry-based proteomics datasets used and/or analyzed during this study are available online at the MassIVE repository (http://massive.ucsd.edu/; direct link: https://doi.org/doi:10.25345/C5SN01G2V; dataset identifier: MSV000095336; Username: “MSV000095336_reviewer”; Password: “ICC-proteomics24"). All WES data is available via the European Genome-Phenome Archive (dataset identifier: EGAD50000001926).

Research involving human participants, their data, or biological material

Policy information about studies with human participants or human data. See also policy information about sex, gender (identity/presentation), and sexual orientation and race, ethnicity and racism.

Reporting on sex and gender	Tumor specimens were included into the cohorts irrespective of a patient's sex and gender, leading to a largely balanced cohorts comprising 45 women and 35 men in the MSKCC-cohort and 33 women and 27 men in the UKF cohort. Subsequent measurements and data analysis were also performed without considering sex and gender as e.g. covariates. Neither the clustering nor the survival outcome was significantly associated to sex and gender. In the PDX proteomic experiment, PDX were chosen regardles of the donor's sex or gender. The xenograft used in the PDX treatment study originated from a male donor.
Reporting on race, ethnicity, or other socially relevant groupings	The patient's race and ethnicity of the MSKCC cohort was documented in the clinical annotation but did not influence the setup of the cohort. Both proteomic clusters include patients from all races and ethnicities, reflecting the ICC patient population at MSKCC at the time of collection. For the UKF cohort, patient's race and ethnicity were not documented. It is noteworthy that the present cohorts include predominantly caucasian patients, while other available proteomic ICC data, including the dataset used to apply the classifier (Dong et al.), originates from East Asian treatment centers. In the PDX proteomic experiment, PDX included xenografts originating from caucasian donors due to their availability. The xenograft used in the PDX treatment study originated from a male caucasian donor.
Population characteristics	The 80 MKSCC patients included present a mean age of 68 years (IQR 61 -73 years). The 62 UKF ICC patients present a mean age of 65 years (IQR 57 - 71 years). All patients presented with an ICC tumor for the first time and were treatment naive. Some patients were previously or concurrently diagnosed with primary sclerosing cholangitis (3 patients) or hepatitis b/c (7 patients), which are risk factors for developing ICC. The PDX stemmed from 9 ICC patients between 47 and 69 years of age. Six of them were female, three were male.
Recruitment	For the first cohort, formalin-fixed, parraffin-embedded patient samples were assembled into the present cohort at the Department for Pathology at the Memorial Sloan Kettering Cancer Center (MSKCC) in New York, USA between 2009 and 2018. The second cohort was assembled at the at the Medical Center – University of Freiburg (UKF) between 2000 and 2022. Besides an established ICC diagnosis, no other parameters were considered. Written inforrmed consent was obtained from all patients.
Ethics oversight	MSKCC institutional review board and UKF Institutional Ethics Committee approved the study (protocol numbers 16-1683A(3) (MSKCC) and 21-1684 (UKF)).

Note that full information on the approval of the study protocol must also be provided in the manuscript.

Field-specific reporting

Please select the one below that is the best fit for your research. If you are not sure, read the appropriate sections before making your selection.

☒ Life sciences ☐ Behavioural & social sciences ☐ Ecological, evolutionary & environmental sciences

For a reference copy of the document with all sections, see nature.com/documents/nr-reporting-summary-flat.pdf

Life sciences study design

All studies must disclose on these points even when the disclosure is negative.

Sample size	No sample size calculations were performed, since sample size was constrained by the availability of patient specimens. The entire available ICC patient population was included. Equally, all available PDX samples were used for the PDX proteomic characterization.
Data exclusions	Six proteomic outliers were reevaluated by a licensed pathologist, reclassified as hepatocellular carcinomas or fibrosis, and removed from the MKSCC patient study. Similarly, four tumor samples were excluded from the UKF patient study, after consultation of a pathologist. No data points were excluded from the PDX proteomic study or the PDX treatment study.
Replication	For patient-material, no replicates were performed due to the scarcity of the material. Classifier results were replicated by bootstrapping, leading to a robust estimate of variables. Cell-line experiments were conducted in triplicates. The PDX animal study was performed with four animals in each treatment group.
Randomization	Pseudonymized patient-samples were randomized into batches for sample preparation and mass-spectrometric measurements. Samples originating from the same patient were assigned to the same digestion batches and measured sequentially. Isogenic PDX mice bearing 75 - 240 mm3 tumors were distributed into experimental groups, with comparable median and mean tumor volumes.
Blinding	Since the patient study was non-interventional, no blinding was performed. For outlier classification, pathologists were blinded to the results of the proteomic study. The assignment of proteomic clusters was performed using unsupervised statistics and thus did not rely on data outside the proteomic results. Cell line experiments and the PDX treatment study were conducted without blinding, since different treatments needed to be applied.

Reporting for specific materials, systems and methods

We require information from authors about some types of materials, experimental systems and methods used in many studies. Here, indicate whether each material, system or method listed is relevant to your study. If you are not sure if a list item applies to your research, read the appropriate section before selecting a response.

Materials & experimental systems

n/a	Involved in the study
☒	☐ Antibodies
☐	☒ Eukaryotic cell lines
☒	☐ Palaeontology and archaeology
☐	☒ Animals and other organisms
☒	☐ Clinical data
☒	☐ Dual use research of concern
☒	☐ Plants

Methods

n/a	Involved in the study
☒	☐ ChIP-seq
☒	☐ Flow cytometry
☒	☐ MRI-based neuroimaging

Eukaryotic cell lines

Policy information about cell lines and Sex and Gender in Research

Cell line source(s)	HuCC-T1 (#JCRB0425) and HuH-28 (#JCRB0426) cell lines were obtained from the Japanese Collection of Research Bioresources Cell Banks (JCRB), and SNU-1079 (#CSC-C9622L) cells were obtained from the Korean Cell Line Bank (KCLB).
Authentication	Cell line authentication was verified by Eurofins Genomics, DNA isolation was carried out from cell pellet and genetic characteristics were determined by PCR-single-locus-technology.
Mycoplasma contamination	All cell lines were tested negative for mycoplasma contamination by Eurofins.
Commonly misidentified lines (See ICLAC register)	We did not use any commonly misidentified cell lines.

Animals and other research organisms

Policy information about studies involving animals; ARRIVE guidelines recommended for reporting animal research, and Sex and Gender in Research

Laboratory animals	Four- to six-week-old female NMRInu/nu mice (Charles River, Germany) were implanted with tumors from human patients under anesthesia.
Wild animals	The study did not involve wild animals.
Reporting on sex	In keeping with the 3R principles, we solely used mice of the same sex for the PDX treatment experiment to yield more homogenous data without further increasing the number of animals. The proteomic ICC PDX data includes human PDX tumors from both sexes.

The PDX treatment study was performed in mice carrying human tumors from a male donor.

Field-collected samples

The study did not involve field-collected samples.

Ethics oversight

This study was carried out in strict accordance with the recommendations in the Guide for the Care and Use of Laboratory Animals of the Society of Laboratory Animals (GV SOLAS). All animal experiments were approved by the Committee on the Ethics of Animal Experiments of the regional council (Permit Numbers: G-20/163 and I-19/02).

Note that full information on the approval of the study protocol must also be provided in the manuscript.

Plants

Seed stocks

not applicable

Novel plant genotypes

not applicable

Authentication

not applicable
